# Polymorphisms in a bacterial signalling pathway alter evolutionary routes of antibiotic resistance in *Escherichia coli*

**DOI:** 10.1371/journal.pbio.3003977

**Published:** 2026-08-26

**Authors:** Chetna Yelpure, Saillesh Chinnaraj, Kush Topiwala, Jay Phadke, Nishad Matange

**Affiliations:** Department of Biology, Indian Institute of Science Education and Research (IISER), Pune, India; Wageningen University, KINGDOM OF THE NETHERLANDS

## Abstract

Antibiotic resistance in bacteria frequently evolves due to mutations in drug-target or detoxifying genes. Pre-existing polymorphisms in these genes are likely to influence the evolvability of drug resistance. However, the role of polymorphisms in driving resistance evolution and the underlying molecular mechanisms are poorly understood. Here, we demonstrate that polymorphisms in a signalling pathway impact the evolvability of trimethoprim (TMP) resistance in *Escherichia coli*. When challenged with TMP, de-repression of the PhoQ-PhoP two-component signalling system in *E. coli* transcriptionally upregulates the drug target Dihydrofolate Reductase (DHFR), leading to drug resistance. We identified and characterised naturally occurring polymorphisms in PhoQ and DHFR that modulate intrinsic antibiotic susceptibility. These variants also altered the ability of *E. coli* to evolve de novo TMP resistance as a result of epistasis with adaptive mutations. Interestingly, a strain harbouring a less-evolvable DHFR variant acquired a novel mutation in PhoQ under TMP pressure that hyperactivated the signalling pathway, conferring high-level resistance but at a large fitness cost. This mutation was not observed in wild type but reached fixation rapidly in the background of the DHFR variant. Using RNA-sequencing we compare how natural variants and adaptive mutations in PhoQ affect the expression of PhoP-target genes and downstream regulatory pathways in *E. coli*. Finally, we uncouple the roles of resistance level by DHFR overproduction and fitness cost by activation of the RpoS regulon to explain why *E. coli* more frequently evolves to de-repress PhoQ than hyperactivate it under drug pressure. Our study, thus, demonstrates that pre-existing polymorphisms alter both, evolvability and mutation landscapes during antibiotic adaptation. This work establishes the PhoQ-PhoP-DHFR pathway as an experimental paradigm to understand the evolution of signalling pathways under environmental selection.

## Author summary

Genetic variation is expected to enhance the evolvability of antibiotic resistance in bacteria. However, the impact of pre-existing genetic polymorphisms on evolvability and underlying mechanisms is poorly understood. In this study, we explore the impact of polymorphisms in the PhoQ-PhoP-DHFR pathway on the evolution of trimethoprim resistance in *Escherichia coli*. We show that polymorphisms in genes of this pathway are epistatic with adaptive mutations. As a result, pre-existing variants alter the outcomes of antibiotic challenge in terms of both resistance evolvability and the landscape of adaptive mutations. We then decipher how naturally occurring and drug-selected mutations in PhoQ affect the global transcriptome of *E. coli* and result in differing extents of pleiotropy. Finally, we uncouple the contribution of fitness cost and benefit of activating the PhoQ-PhoP signalling system during evolution and define drug concentration regimes in which different activity levels are likely to be favoured.

## Abstract 3

Why some bacteria evolve antibiotic resistance more readily than others is poorly understood. This study shows that naturally occurring single nucleotide polymorphisms in the PhoQP system changes how readily E. coli evolves trimethoprim resistance and which mutations enable it.

## Introduction

Antimicrobial resistance (AMR) is the ability of bacteria to overcome growth inhibition by antibiotics. An increasing burden of AMR threatens to destabilise decades of medical advance and understanding its evolution may hold the key to slowing it down [1,2]. In addition to its biomedical relevance, antibiotic resistance is also an important model for understanding molecular, genetic, and evolutionary mechanisms of bacterial adaptation. Moreover, it has been used to study evolvability itself [3]. Evolvability was originally defined as the disposition of a biological system to generate and maintain variation. More recently, it is equated with adaptability, i.e., degree to which a biological system can undergo adaptive evolution [3–10]. The latter definition is particularly relevant for antibiotic resistance as predicting the potential for resistance evolution is key to mitigating its adverse effects.

Three processes dictate evolvability [3,8]. First, the generation of genetic variation, second, genotype-to-phenotype mapping of variants; and third, selection by the environment. In the case of adaptation to antibiotics, changes in evolvability have been traced to all three steps. Hypermutator strains that generate greater genetic variation emerge spontaneously during antibiotic challenge and have an enhanced capacity for adaptation to antibiotics [11–13]. Conversely, inhibiting transcription-coupled mutagenesis can slow down the evolution of drug resistance [14,15]. Likewise, epistasis among resistance-conferring mutations alters genotype-phenotype mapping and fitness, directly influencing resistance evolution [16,17]. Yet, empirical and experimental evidence linking these three processes and investigating how they affect one another is scarce. For instance, while it is intuitive that greater pre-existing genetic variation would tend to increase evolvability, how pre-existing polymorphisms alter the fitness landscape of adaptive mutations and, consequently, outcomes of antibiotic challenge is less well worked out.

A few studies have demonstrated the role of pre-existing sequence variants in antibiotic resistance. For instance, the MacLean group showed that mixed strain *Pseudomonas* infections had a higher propensity to develop antibiotic resistance [18]. Similarly, pre-existing chromosomal polymorphisms enhanced the evolvability of *Escherichia coli* towards colistin [19]. In some cases, molecular and genetic mechanisms have also been suggested. For example, epistasis between pre-existing variants and resistance-conferring mutations has been shown to enhance evolvability of drug resistance in *Neisseria*, *Streptococcus* and *Staphylococcus* through different molecular mechanisms like metabolism, drug efflux and cyclic nucleotide signalling [20–22]. Polymorphisms in bacterial two-component systems impact multiple phenotypes. For instance, polymorphisms in two-component systems affect traits such as quorum sensing (ComP/ComA in *Bacillus* [23]), pathogenesis (RocA/CsrR/CsrS in *Streptococcus* [24]) and pH sensing (ArsS in *Helicobacter* [25], EvgS in *E. coli* [26]). However, though the molecular basis of these phenotypes is understood, their impact over evolutionary timescales is unknown. In the present study, we bridge this gap by linking molecular mechanistic impacts of polymorphisms in a bacterial signalling pathway with their contribution to evolvability of drug resistance.

**Fig 1:**
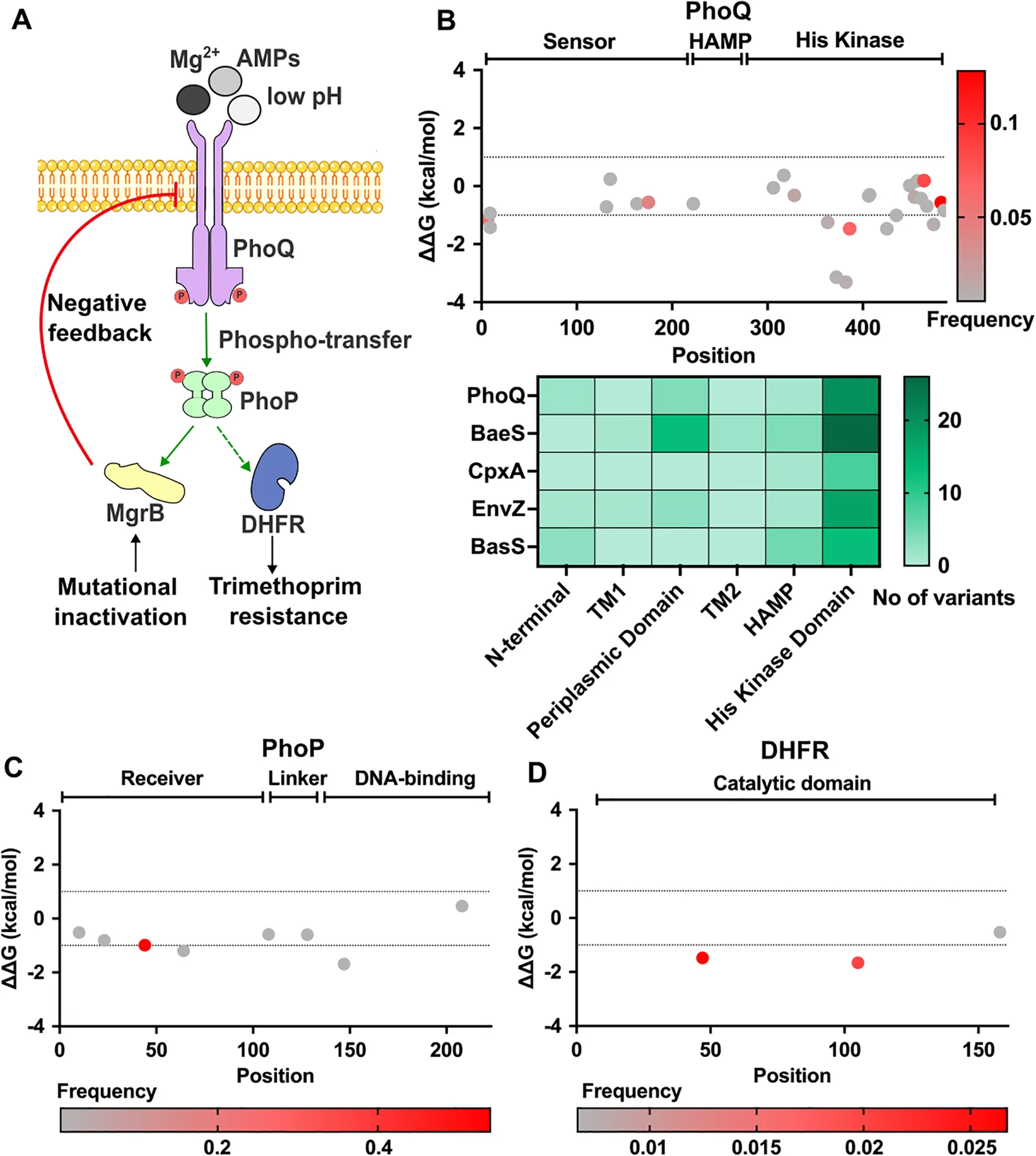
Polymorphisms in the PhoQ-PhoP-DHFR pathway of *E. coli.* **A:** Schematic representation of the PhoPQ two-component system and its target DHFR implicated in resistance to TMP in *E. coli*. Active PhoQ results in phosphorylation of the response regulator PhoP. PhoP positively regulates transcription of *mgrB* and *folA* (coding for the DHFR enzyme). MgrB inhibits the kinase activity of PhoQ, setting up a negative feedback loop. Evolution under TMP pressure results in mutational inactivation of *mgrB* leading to PhoQ de-repression. This increases DHFR transcription and confers trimethoprim resistance to *E. coli*. **B:** *Top panel* Distribution of naturally occurring polymorphisms in the PhoQ protein from *E. coli*. Position of each polymorphism in PhoQ (X-axis) and predicted stability (ΔΔG, Y-axis) are plotted. Values of ΔΔG between −1 and +1 kcal/mol were considered to be neutral. The frequency of occurrence of each variant is represented by a grey-to-red colour scale as shown. Domain boundaries are indicated above the plot. *Bottom panel:* Domain-wise distribution of polymorphisms in PhoQ and 4 different sensor kinases from *E. coli*. Enrichment of variants in the kinase domain is apparent in all cases. **C, D:** Distribution of naturally occurring polymorphisms in the PhoP (C) and DHFR (D) proteins from *E. coli*. As with PhoQ, position of each polymorphism (X-axis) and predicted stability (ΔΔG, Y-axis) are plotted. The frequency of occurrence of each variant is represented by a grey-to-red colour scale as shown. Domain boundaries are marked above the plot. The data underlying this Figure can be found in S1 File.

In earlier work [27], we discovered a gene regulatory pathway responsible for adaptation of *Escherichia coli* to Trimethoprim (TMP) (Fig 1A). TMP is a broad-spectrum antibiotic that inhibits Dihydrofolate Reductase (DHFR), encoded by the *folA* gene. The susceptibility of *E. coli* to TMP is contingent on the expression level of DHFR [28]. Upon TMP challenge, *E. coli* upregulates the expression of *folA* via *cis-*regulatory mutations in the promoter of *folA* or higher gene copy number [29]. However, one of the earliest loci implicated is *mgrB*, coding for a small membrane protein that inhibits the PhoQ-PhoP two-component signalling system. Mutations in *mgrB* that emerge spontaneously under TMP pressure either inactivate the gene or its promoter [27,30]. In the absence of MgrB, the PhoQ sensor kinase is de-repressed, resulting in greater phosphorylation of PhoP, its cognate response regulator. An increase in active PhoP enhances the expression of DHFR and improves fitness of *E. coli* in the presence of TMP (Fig 1A) [27,30].

Though inactivation of MgrB results in low-level TMP resistance by itself, it synergizes with mutations in DHFR that arise subsequently to confer high-level drug resistance [27,30]. As a result, loss of MgrB acts as a gateway mutation that facilitates the evolution of TMP resistance in *E. coli*, such that preventing mutations in *mgrB* slows down the evolution of resistance [30]. The PhoQ-PhoP-DHFR pathway, therefore, provides an ideal system to investigate the impact of pre-existing genetic variation on the evolution of drug resistance. In this study, we combine bioinformatic data mining, allele reconstruction, structure-based mutagenesis, and experimental evolution to uncover genetic polymorphisms in PhoQ and DHFR that alter the intrinsic TMP susceptibility of *E. coli*, as well as the potential to evolve resistance. Using transcriptomics and co-culture experiments on natural variant and drug-selected mutants we ask how TMP concentration determines the optimal activity of PhoQ during adaptation. Finally, we uncouple the relative contributions of benefit due to increased DHFR and cost due to cross-activation of other transcriptional programs in determining PhoQ activity during evolution.

## Results

### Sequence variation within the PhoQ-PhoP-DHFR pathway among *E. coli* strains

To assess the extent of genetic polymorphism within the PhoQ-PhoP-DHFR pathway (Fig 1A), we compared their sequences across strains of *E. coli*. A total of 180, 204, and 150 protein sequences of PhoQ, PhoP, and DHFR, respectively, from strains catalogued in the NCTC3000 database [31] were aligned and polymorphisms were identified by comparing with *E. coli* K-12 MG1655 (S1 File). Since this database contains many historically archived strains, we expected most polymorphisms to have emerged in the absence of antibiotic pressure. Commensurate with its large size, PhoQ, the sensor histidine kinase, showed the greatest number of sequence variants at 28 polymorphic sites (Fig 1B). PhoP had an intermediate number of polymorphisms at 8 sites (Fig 1C) while DHFR was the most conserved with only 3 polymorphic sites (Fig 1D). MgrB showed 6 polymorphisms (S1 File). However, it also showed several frame-shift variants similar to those isolated from trimethoprim resistant *E. coli* [27,30].

For PhoQ, the largest number of polymorphisms were in its C-terminal histidine kinase domain (Fig 1B). Interestingly, an enrichment of polymorphisms within the kinase domain was also observed in other sensors such as BaeS, CpxA, EnvZ, and BasS, all of which are implicated in antibiotic resistance [32–36], indicating a general rather than PhoQ-specific trend (Fig 1B, S1 File). We next computationally predicted the ΔΔG values of folding of all variants compared to wild type (S1 File) [37]. Since functional changes are often associated with perturbation of structure and function, exemplified by studies on DHFR [38–40], we used these predictions as a proxy for activity. All sequence variants of PhoP, except one, were predicted to be neutral (i.e., predicted ΔΔG between −1 and +1 kcal/mol) (Fig 1C). Similarly, most PhoQ variants were also predicted to be near-neutral (Fig 1B). However, Val_382_Gly and Phe_372_Gly, both located in the kinase domain of PhoQ, were predicted as highly destabilising (ΔΔG of −3.3 and −3.1 kcal/mol, respectively). Two of the three variants of DHFR, i.e., Trp_47_Arg and Pro_105_Ala were also predicted to be destabilising (ΔΔG of −1.48 and −1.66 kcal/mol, respectively) (Fig 1D). Based on these analyses we surmised that proteins of the PhoQ-PhoP-DHFR pathway demonstrated different extents of sequence polymorphism and some variants, such as those in PhoQ and DHFR, may have consequences for TMP susceptibility of *E. coli*.

### Val_382_Gly is a mildly activating polymorphism in PhoQ that facilitates the evolution of resistance

**Fig 2:**
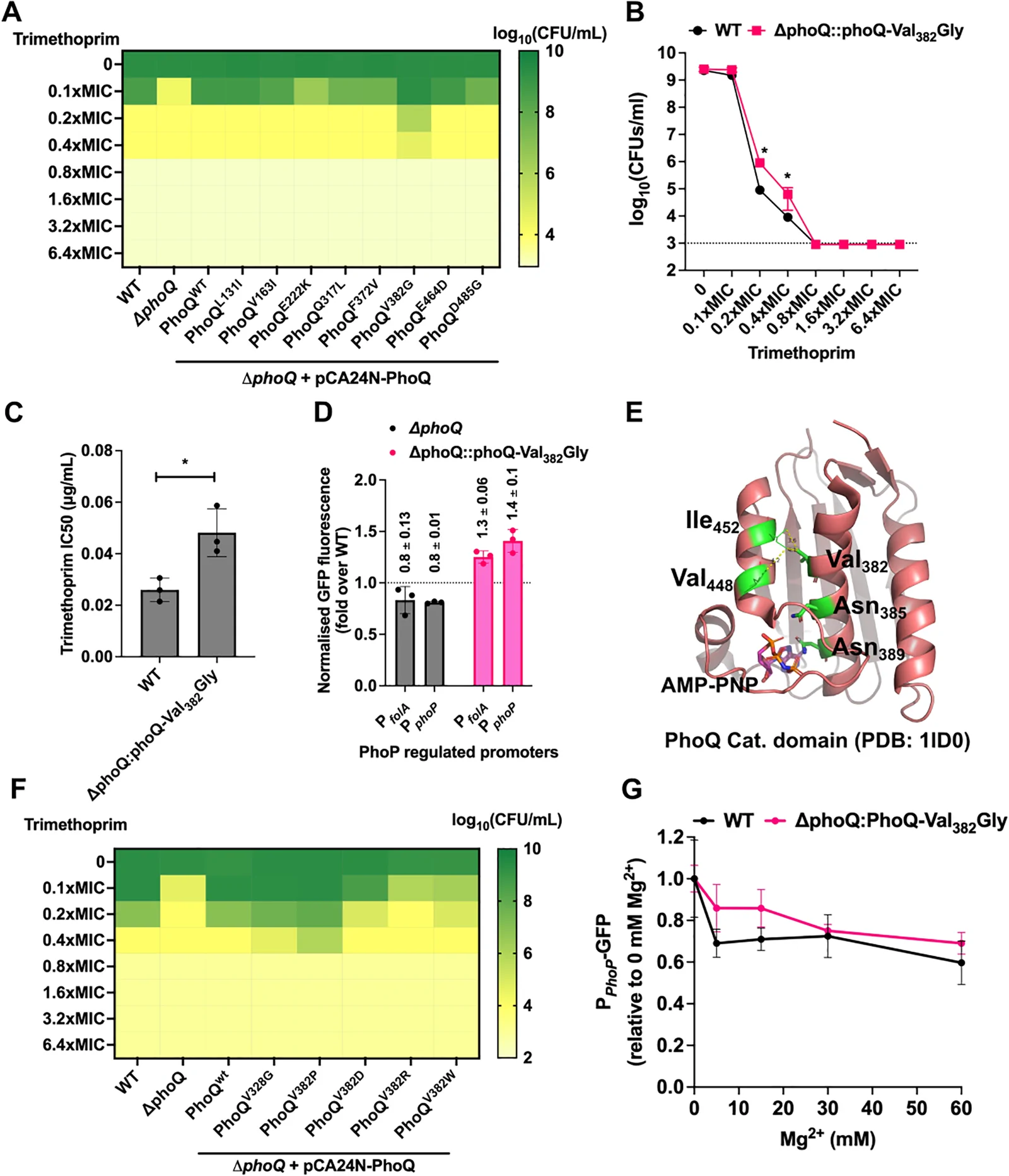
Val_382_Gly is a mildly activating polymorphism in PhoQ. **A:** Colony-forming efficiencies of 8 naturally occurring PhoQ variants at different trimethoprim concentrations. Individual sequence variants of PhoQ were introduced into a *phoQ* knockout strain on a multi-copy plasmid. Mean log_10_(CFU/mL) values from 3 independent experiments are plotted on a yellow-green colour gradient. Individual raw values are provided in S2 File. **B:** Colony forming efficiency of wild type (black) and the PhoQ-Val_382_Gly knock-in (magenta) strains across trimethoprim concentrations. Mean log_10_(CFU/mL) values ± SD from three replicates are plotted (*p-value<0.05, Welch’s *t* test). **C:** Trimethoprim IC50 values for wildtype and PhoQ-Val_382_Gly knock-in strains. Mean ± SD from 3 replicates is plotted as bars and individual values are shown (*p-values≤0.05, Welch’s *t* test). **D:** Promoter activity of P*phoP* and P*folA* in the *phoQ* knockout (black) and PhoQ-Val_382_Gly knock-in strains (magenta). Promoter activity was measured as the ratio of GFP reporter fluorescence to OD at 600 nm and normalised to wild type (set to 1, marked by a dotted line). Mean ± SD of 3 replicates and individual data points are plotted (*p-values≤0.05, Welch’s t *t*est). **E:** Crystal structure of the catalytic histidine kinase domain of PhoQ (PDB: 1ID0) is shown as a cartoon. Bound ATP-analog AMPPNP is shown as sticks and coloured by element (N: Blue; C: Magenta, O: Red, P: Orange). Residue Val_382_ in the ATP-binding helix, possible interacting residues Ile_452_ (3.6 Å) and Val_448_ (4.2 Å) and N-box residues (Asn_385_ and Asn_389_) are shown as sticks and coloured by element (C: Green; N: Blue; O:Red). **F:** Colony-forming efficiencies for different substitutions at Val_382_ in PhoQ. Mean log_10_(CFU/mL) values from 3 independent experiments are plotted on a yellow-green colour gradient. Individual raw values are provided in S2 File. **G:** P*phoP* promoter activity in *E. coli* wild type (black) and PhoQ-Val_382_Gly knock-in (magenta) strains over a range of Mg^2+^ concentrations. Promoter activity at each concentration of Mg^2+^ was normalised to activity in the absence of Mg^2+^ supplementation. Mean ± SD from 3 replicates is plotted. The data underlying this Figure can be found in S2 File.

Given the large number of variants in PhoQ, we first asked whether naturally occurring sequence polymorphisms in this protein had phenotypic consequences for *E. coli*. We selected 8 polymorphisms based on their location in PhoQ and expressed them from a plasmid in *E. coli ΔphoQ*. PhoQ-Val_382_Gly enhanced colony formation on TMP-containing media, while the other 7 variants had near-neutral phenotypes (Fig 2A, S2 File). To rule out artefacts due to plasmid-based expression, we generated a scarless genomic knock-in of PhoQ-Val_382_Gly at its native chromosomal locus. Indeed, a small but significant increase in colony forming efficiency on TMP-supplemented agar (Fig 2B) and 1.6-fold higher IC50 (concentration required for 50% growth inhibition) in broth cultures (Fig 2C) was observed for the knock-in strain. Further, the promoter activity of *PphoP* and *PfolA*, both responsive to active PhoP [27,41], was higher by 40% and 30%, respectively, in the knock-in compared to wild type (Fig 2D). Taken together, these results identified Val_382_Gly to be a mildly activating, naturally occurring polymorphism in PhoQ.

Val_382_ is located in an alpha-helix proximate to the conserved ‘N box’ in the catalytic domain, which interacts with the α-phosphate of ATP (Fig 2E) [42]. To better understand why Val_382_Gly led to activation, we created other amino acid substitutions at this site that altered flexibility, polarity, size and charge. Of all the substitutions generated, only Pro and Gly, enhanced TMP resistance (Fig 2F, S2 File). On the other hand, substitution of Val_382_ with Arg or Trp reduced colony formation on TMP-supplemented media, while substitution with Asp resembled wild type (Fig 2F, S2 File). Since Pro and Gly are helix-breaking amino acids due to their distinct structural properties, we reasoned that loss of local helicity or enhanced flexibility of this region was likely to activate the kinase domain of PhoQ. Importantly, like the wild type receptor, PhoQ-Val_382_Gly continued to be inhibited by Mg^2+^ ions, which bind to the extracellular sensor domain and allosterically inhibit the catalytic domain [43] (Fig 2G). These results indicated that while Val_382_Gly resulted in higher basal activity due to a structural perturbation of the kinase domain, it did not alter signal transduction between the domains of PhoQ.

**Fig 3:**
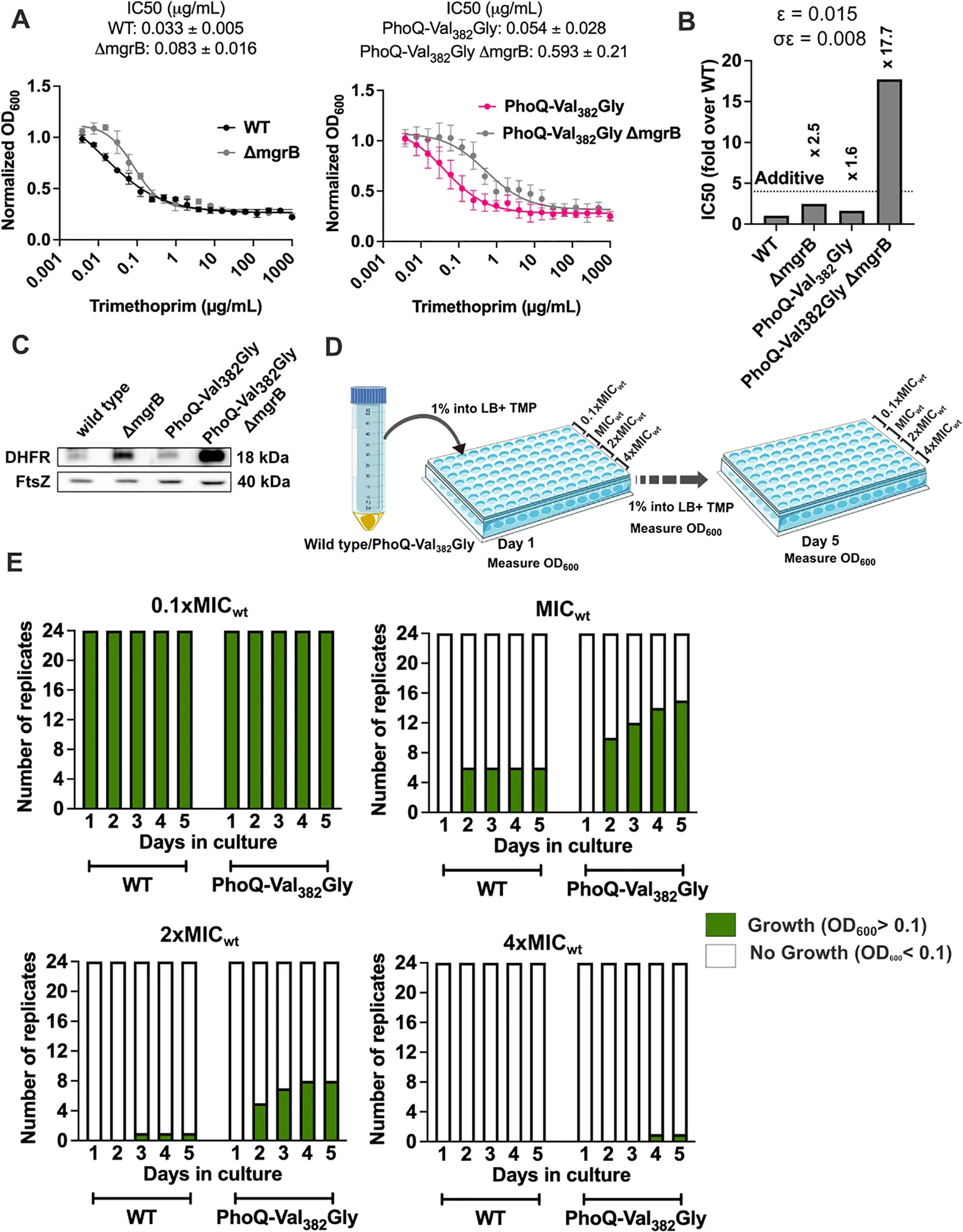
PhoQ-Val_382_Gly facilitates the evolution of trimethoprim resistance. **A:** *Left panel.* Trimethoprim dose response for wild type (black) and *ΔmgrB* (grey) strains of *E. coli*. *Right panel.* Trimethoprim dose response for PhoQ-Val_382_Gly knock-in strain (magenta) and its *ΔmgrB* derivative (grey). Growth at each concentration of TMP is normalised to growth in drug-free media. Mean ± SD from 3 replicates is plotted. Values of IC50 derived from curve-fitting (mean ± SD) are provided above the graph. **B:** Synergistic epistasis between PhoQ-Val_382_Gly and *ΔmgrB* alleles. Fold IC50 over wild type for indicated strains are plotted as bars and values are provided. The values of ε and σε indicating positive epistasis are shown above the bars. The expected fold change based on an additive interaction is indicated by a dotted line. **C:** DHFR expression in *E. coli* wild type, *ΔmgrB*, PhoQ-Val_382_Gly knock-in and its *ΔmgrB* derivative assessed using immunoblotting. FtsZ was used as a loading control. Representative data from 3 independent replicates are shown. **D:** Schematic for the antibiotic challenge assay used to assess evolvability of trimethoprim resistance for *E. coli* wildtype and the PhoQ-Val_382_Gly knock-in strains. **E:** Number of replicates of *E. coli* wildtype and the PhoQ-Val_382_Gly knock-in strains showing growth over 5 days of antibiotic challenge at different TMP pressures. The data underlying this Figure can be found in S2 File and S1 Raw Image.

Strikingly, deleting *mgrB* synergised with PhoQ-Val_382_Gly to confer high level resistance to TMP (Fig 3A). Indeed, PhoQ-Val_382_Gly and *ΔmgrB* together resulted in ~17 fold higher IC50 of TMP compared to ~2.5 fold for *ΔmgrB* alone (Fig 3A and 3B), demonstrating strong positive epistasis (ε = 0.015, σε = 0.008) between these two alleles. The difference arose due to higher DHFR expression in the PhoQ-Val_382_Gly *ΔmgrB* strain, showing that PhoQ-Val_382_Gly led to more profound activation of the signalling pathway upon de-repression (Fig 3C). This result suggested that even though PhoQ-Val_382_Gly was itself only mildly activating, it could facilitate the evolution of high level resistance to TMP. In order to directly test this premise, we checked for the evolvability of the knock-in strain using a drug challenge assay. Here, 24 replicate populations each of wild type and the PhoQ-Val_382_Gly knock-in were subjected to 4 different TMP concentrations, i.e., 100 ng/mL (0.1xMIC_wt_), 1 μg/mL (MIC_wt_), 2 μg/mL (2x MIC_wt_) and 4 μg/mL (4x MIC_wt_), over a period of 5 days. At the end of each day, we passaged 1% of all populations into fresh growth media and assessed the number of surviving replicates of each genotype (Fig 3D). Evolvability was estimated as the number of replicates that evolved resistance, i.e., by estimating extent of adaptation. As expected, at MIC_wt_ and above the wild type showed a dramatic loss in viability, and though a few populations did evolve resistance, a majority were driven to extinction (Fig 3E). Interestingly, a greater number of replicates of the PhoQ-Val_382_Gly knock-in survived the drug challenge and evolved resistance under drug pressure (Cox proportional-hazards ratio at MIC_wt_ β1 = 3.27, 95% CI = 1.342 to 9.160", likelihood ratio = 6.948, *p* = 0.0084) (Fig 3E). Thus, the PhoQ-Val_382_Gly polymorphism, though only mildly activating, enhanced the evolvability of *E. coli* under TMP pressure.

### A hypersensitive variant of DHFR with reduced evolvability reveals a novel activating mutation in PhoQ

**Fig 4:**
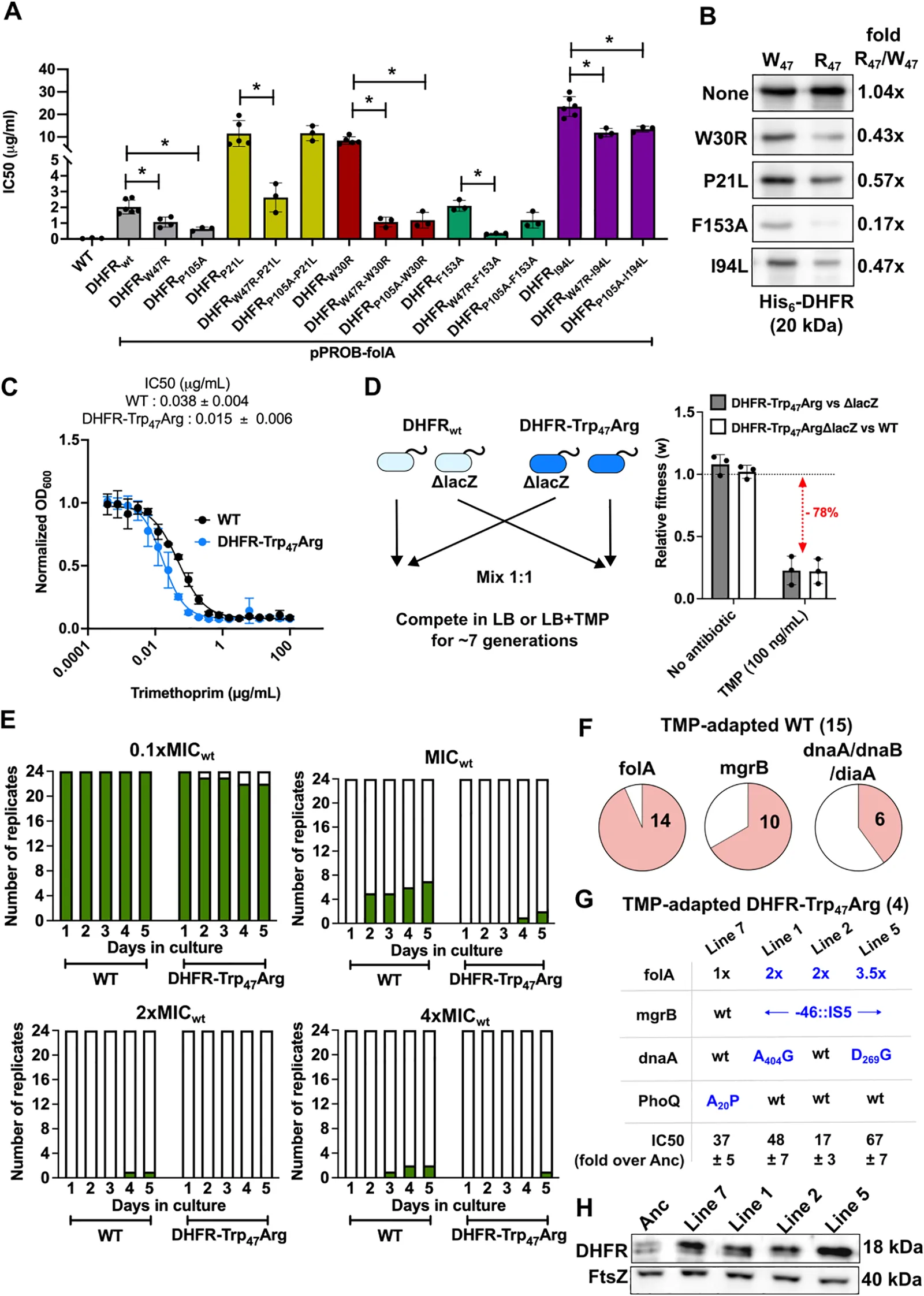
DHFR-Trp_47_Arg knock-in is TMP hypersensitive and evolves resistance via a novel mutation in PhoQ. **A:** Trimethoprim IC50 values for *E. coli* strains heterologously expressing natural DHFR variants, resistant mutants and their combinations. Bars represent mean ± S.D. from at least 3 independent replicates. Individual values are also plotted (*p-values≤0.05, Welch’s *t* test). **B:** Expression level of heterologously expressed DHFR harbouring resistance-conferring mutation with and without Trp_47_Arg assessed by immunoblotting. Representative data from 3 independent replicates in shown. Ratio of expression level of each resistant mutant with and without Trp_47_Arg mutation is provided (fold R_47_/W_47_). **C:** Trimethoprim dose response for *E. coli* wild type (black) and the DHFR-Trp_47_Arg knock-in (blue) strains. Growth at each concentration of TMP is normalised to growth in drug-free media. Mean ± SD from 3 replicates is plotted. Values of IC50 derived from curve-fitting (mean ± SD) are provided above the graph. **D:** *Left panel*. Schematic for measurement of relative fitness of the DHFR-Trp_47_Arg knock-in strain. *Right panel*. Relative fitness of the DHFR-Trp_47_Arg knock-in strain in the absence or presence of TMP. Empty bars represent data from competitions between *E. coli ΔlacZ* and the DHFR-Trp_47_Arg knock-in, while solid bars represent competitions between *E. coli* wild type and the *ΔlacZ* derivative of the DHFR-Trp_47_Arg knock-in. Bars represent mean ± SD from 3 replicates. Individual data points are also plotted. No change in fitness (w = 1) is shown as a dotted line. Magnitude of cost upon addition of trimethoprim to growth media is also shown. **E:** Evolvability of *E. coli* wild type and the DHFR-Trp_47_Arg knock-in strain assessed using an antibiotic challenge assay. Number of replicates of each genotype showing growth over a period of 5 days at different TMP pressures are plotted. **F:** Prevalence of mutations in *folA* (gene duplication/single nucleotide changes), *mgrB* and *dnaA/dnaB/diaA* in 15 replicate evolving lineages of *E. coli* wild type subjected to 500 ng/mL of TMP after 12 passages. Number of replicate lineages in which mutations in each of the indicated genes were detected are shown as pie charts. **G:** Mutations at the *folA*, *mgrB*, *dnaA* and *phoQ* loci detected in the 4 surviving lineages of DHFR-Trp_47_Arg knock-in strain that had adapted to TMP. **H:** Immunoblot to assess the expression level of DHFR in the ancestral DHFR-Trp_47_Arg knock-in strain and 4 surviving lineages that were passaged in TMP. FtsZ was used as a loading control. Representative data from 3 replicates are shown. The data underlying this Figure can be found in S1 Raw Image and S2 and S3 Files.

We next turned our attention to the two polymorphisms in DHFR that were predicted to alter function, i.e., Trp_47_Arg and Pro_105_Ala. When expressed from a plasmid, both variants conferred a 30%–40% lower IC50 than wild-type DHFR (Fig 4A). Intrigued by the finding, we next sought to study the effect of combining these polymorphs with previously reported trimethoprim-resistant mutations in DHFR since intragenic epistasis has been reported for this protein [38,39,44]. Strikingly, combining Trp_47_Arg with at least 4 different resistance-conferring mutations led to substantially diminished TMP resistance (Fig 4A). A similar impact was observed with Pro_105_Ala, though the magnitude of this effect was milder (Fig 4A). Since Trp_47_Arg showed a more pronounced phenotype, we proceeded with this variant for further experiments. A trade-off between in vivo proteolytic stability and resistance has been reported for DHFR and is the molecular basis for negative epistasis among resistance conferring mutations in the protein [38,39]. To test if this mechanism also explained the impact of Trp_47_Arg, we analysed the expression level of plasmid-borne DHFR harbouring each of the 4 resistance conferring mutations, with and without the natural variant. Indeed, Trp_47_Arg exacerbated the proteolytic instability of resistant DHFR alleles in cells, leading to reduced expression levels, and explaining the observed negative epistasis (Fig 4B).

Next, we generated a scarless knock-in of DHFR-Trp_47_Arg at the native *folA* locus. The knock-in strain showed a ~2-fold reduction in TMP IC50 (Fig 4C). It also showed a lower relative fitness than wild type in the presence of TMP (100 ng/mL), but not in drug-free media (Fig 4D), establishing it as hypersensitive to TMP. Given its negative epistasis with resistance-conferring mutations (Fig 4A), we investigated whether DHFR-Trp_47_Arg compromised evolvability of *E. coli* under TMP pressure. Indeed, the DHFR-Trp_47_Arg knock-in strain had a consistently lower evolvability at high drug pressure (Cox proportional-hazards ratio at MIC_wt_ β1 = 0.1504, 95% CI = 0.007961 to 0.8825, likelihood ratio = 4.482, *p* = 0.0343), and also led to a few replicates from 0.1xMIC_wt_ to go extinct (Fig 4E). Thus, DHFR-Trp_47_Arg induced hypersensitivity to TMP and also compromised the evolvability of *E. coli* under TMP pressure.

We asked whether the DHFR-Trp_47_Arg knock-in was at all capable of evolving TMP resistance and, if so, whether it adapted through similar mutations as the wild type. To facilitate adaptation, we performed a serial-transfer based laboratory evolution experiment using 15 replicate populations each of wild type and the DHFR-Trp_47_Arg knock-in. Both genotypes were exposed to 500 ng/mL of TMP (MIC_wt_/2) for 12 growth cycles. Evolution experiments were performed in 3 sets of 5 replicates each, to minimise the chance of cross-contamination of replicates. As expected, at the end of 12 passages, all replicate populations of the wild type showed improved growth in TMP, though most replicates of the DHFR-Trp_47_Arg knock-in went extinct. However, 4 replicates survived and showed enhanced growth in TMP-supplemented media. Whole-genome sequencing revealed that TMP-adapted wild-type populations predominantly harboured inactivating mutations in *mgrB,* and point mutations or duplications in *folA* (Fig 4F, S3 File). Additionally, mutations in *dnaA* and its interacting proteins *dnaB* and *diaA* were also found repeatedly (Fig 4F, S3 File). Though some other loci also harboured mutations, at least two of the above genes (i.e. *folA*, *mgrB* and *dnaA/dnaB/diaA*) were mutated in all 15 replicates, implicating them as the major determinants of TMP resistance. This convergent pattern of mutations could not be attributed to artefacts such as cross-contamination, as even though the same loci were implicated across replicates, the precise mutations, as well as co-occurring mutations in other genes, were not identical (S3 File). Three out of 4 surviving populations of DHFR-Trp_47_Arg showed a similar mutational landscape, i.e., loss-of-function mutations in *mgrB* and amplification of the *folA* gene (Fig 4H, S3 File). Two of these three replicates also harboured mutations in *dnaA* (Fig 4H, S3 File). However, the fourth surviving population did not have mutations in *mgrB, folA* or *dnaA*. Instead, it harboured a single mutation, Ala_20_Pro, in the PhoQ sensor kinase (Fig 4H, S3 File). This mutation was absent from the list of naturally occurring PhoQ polymorphisms (S1 File). However, a single report identifying the same mutation from a clinical isolate of chlorhexidine/colistin resistant *Klebsiella pneumoniae* suggesting that it may be relevant under antibiotic pressure [45]. Interestingly, while *mgrB* mutations and *folA* amplifications co-occurred in the other three TMP-evolved populations, PhoQ-Ala_20_Pro was a stand-alone mutation (Fig 4H, S3 File). Nonetheless, it had elevated DHFR expression levels and showed comparable IC50 values to the other evolved populations (Fig 4G).

### Substitution of Ala_20_ with Pro hyperactivates PhoQ

**Fig 5:**
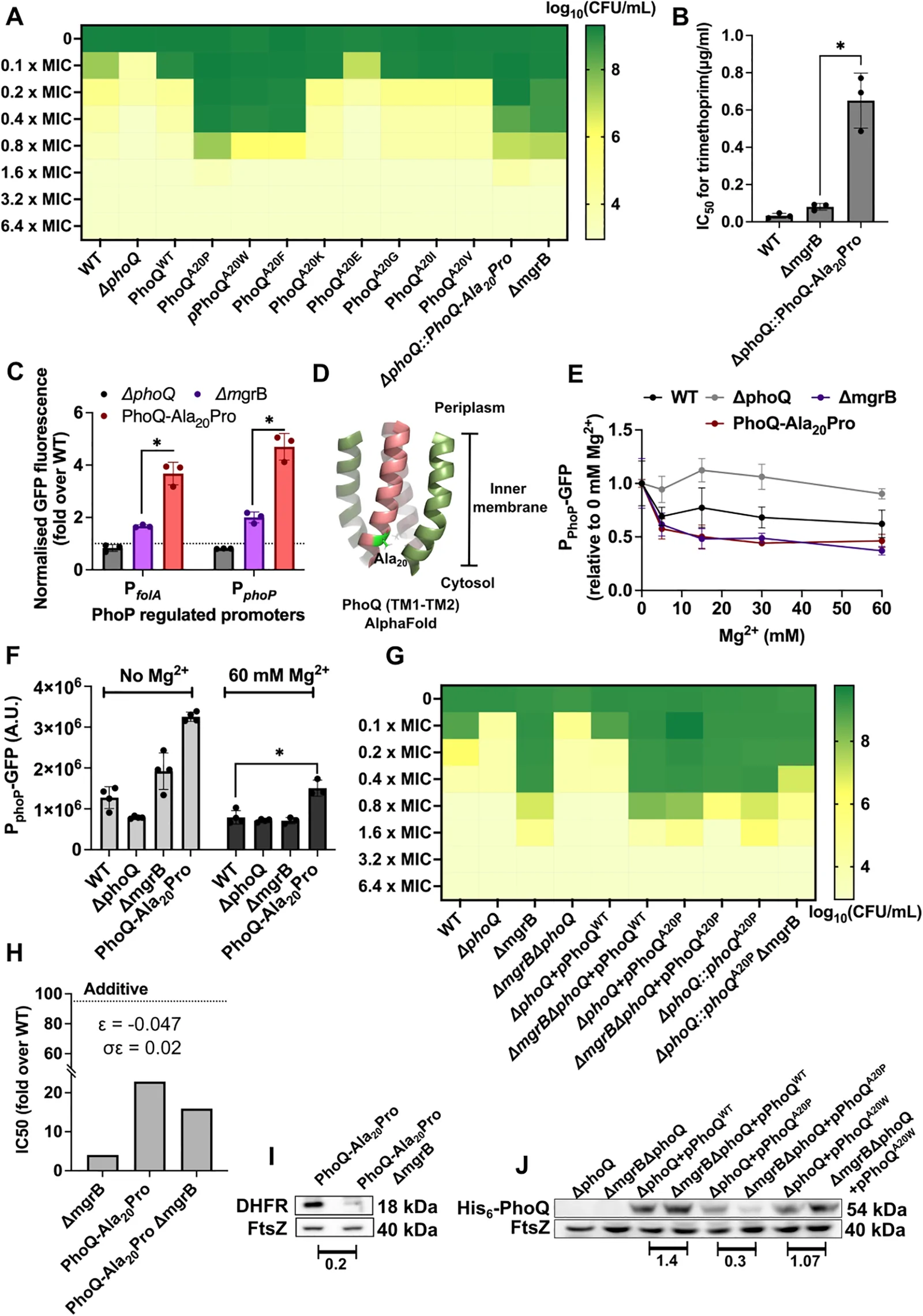
Ala_20_Pro mutation hyperactivates the PhoQ Kinase and alters the impact of allosteric regulators. **A:** Colony-forming efficiencies of various mutations at Ala_20_ in PhoQ at different trimethoprim concentrations. Individual mutants of PhoQ were introduced into a *phoQ* knockout strain on a multi-copy plasmid. The last two columns represent colony forming efficiencies of the PhoQ-Ala20Pro knock-in (*ΔphoQ::PhoQ-Ala*_*20*_*Pro)* and *ΔmgrB* strains. Mean log_10_(CFU/mL) values from 3 independent experiments are plotted on a yellow-green colour gradient as indicated. Individual raw values are provided in S2 File. **B:** Trimethoprim IC50 for *E. coli* wild type, *ΔmgrB* and PhoQ-Ala_20_Pro knock-in strains. Mean ± SD from 3 replicates and individual data points are plotted (*p-value ≤0.05; Welch’s *t* test). **C:** Promoter activity for P*phoP* and P*folA* in *E. coli ΔphoQ, ΔmgrB* and PhoQ-Ala_20_Pro knock-in strains, measured as GFP fluorescence/OD_600_ and normalised to wild type (set to 1). Mean ± SD from 3 replicates and individual data points are plotted (*p-value ≤0.05; Welch’s *t* test). **D:** Transmembrane region (helices H1 and H2) from a model of the PhoQ dimer generated using Alphafold. Helix H1 is shown in tan and H2 is shown as an olive cartoon. Ala_20_ is represented as sticks and coloured by element (C: Green). **E:** Promoter activity of P*phoP* in the presence of different concentrations of Mg^2+^ in *E. coli* wild type, *ΔphoQ, ΔmgrB* and PhoQ-Ala_20_Pro knock-in strains. Promoter activity at each concentration of Mg^2+^ was normalised to activity in the absence of Mg^2+^ supplementation. Mean ± SD from 3 replicates is plotted as bars. **F:** Promoter P_*phoP*_ activity at 0 and 60 mM of Mg^2+^. GFP fluorescence was normalised to growth (OD at 600nm). Mean ± SD from at least 3 replicates for each strain is plotted as bars. Individual data points are also shown (*p-value ≤0.05; Welch’s *t* test). **G:** Epistasis between *ΔmgrB* and PhoQ-Ala_20_Pro alleles. Colony-forming efficiencies for PhoQ or PhoQ-Ala_20_Pro expressed from a plasmid (pPhoQ^WT^/pPhoQ^A20P^) in indicated genetic backgrounds, or the PhoQ-Ala_20_Pro knock-in strain (*ΔphoQ::PhoQ-Ala*_*20*_*Pro*) with and without *mgrB* at different TMP concentrations. Mean log_10_(CFU/mL) from 3 replicates is plotted on a yellow-green colour scale. Individual raw data are available in S2 File. **H:** Negative epistasis between *ΔmgrB* and PhoQ-Ala_20_Pro alleles measured using fold change in IC50 of trimethoprim (over wild type) for the genomic knock-in of PhoQ-Ala_20_Pro with and without *mgrB*. The values of ε and σε indicating negative epistasis are shown above the bars. Fold change in IC50 values based on an additive interaction is marked with a dotted line. **I:** DHFR expression level in the PhoQ-Ala_20_Pro knock-in strain and its *ΔmgrB* derivative assessed using immunoblotting. FtsZ was used as a loading control. Representative data from 3 replicates is shown. Fold change in expression upon deletion of *mgrB* is given below the immunoblot. **J:** Expression level of PhoQ protein determined using immunoblotting with an anti-HexaHis antibody. Wild type PhoQ or its mutants was expressed heterologously in the indicated genetic backgrounds. FtsZ was used as a loading control. Fold change for between *mgrB*-deficient and *mgrB*-expressing backgrounds is provided below the immunoblot. Representative data from 3 independent replicates is shown. The data underlying this Figure can be found in S2 File and S1 Raw Image.

Since PhoQ-Ala_20_Pro was detected in the background of DHFR-Trp_47_Arg, we first asked whether the TMP resistant phenotype was due to a combined effect of the two alleles. We generated a plasmid-borne copy of PhoQ-Ala_20_Pro, introduced it into *E. coli ΔphoQ* (which had a wild-type *folA* locus) and found that it conferred high level of TMP resistance (Fig 5A, S2 File). We also generated a scarless genomic knock-in of PhoQ-Ala_20_Pro. This strain was resistant to TMP as well (Fig 5A and 5B, S2 File). Thus, PhoQ-Ala_20_Pro conferred TMP resistance independent of polymorphisms at the *folA* locus. The PhoQ-Ala_20_Pro knock-in showed substantially higher promoter activity of *PfolA* and *PphoP* compared to wild type (Fig 5C). Indeed, both promoters were stimulated to a greater extent than by MgrB deficiency (Fig 5C), demonstrating that PhoQ-Ala_20_Pro hyperactivated the two-component system beyond its de-repressed state. In line with this result, the IC50 of TMP for the PhoQ-Ala_20_Pro knock-in was higher than *E. coli ΔmgrB* (Fig 5B).

Ala_20_ lies in the transmembrane helical region (TM1 helix) of PhoQ close to the cytosolic face of the inner membrane (Fig 5D) [46]. Since proline mutations introduce kinks in alpha-helices, we wondered if this might explain high activity of PhoQ-Ala_20_Pro. Like with Val_382_Gly, we generated a series of substitutions at Ala_20_ and tested their phenotype. Unexpectedly, PhoQ-Ala_20_Gly, which could also alter helicity, phenotypically resembled wild-type PhoQ (Fig 5A, S2 File). Substituting Ala_20_ with a negatively charged amino acid, i.e., Glu, resulted in TMP sensitivity compared to the wild type, while positively charged Lys phenocopied wild-type PhoQ (Fig 5A, S2 File). Interestingly, introduction of aromatic amino acids Trp or Phe, but not aliphatic amino acids Ile or Val, conferred TMP resistance, albeit to a lower extent than Pro (Fig 5A, S2 File). Thus, helix kinking or increased local flexibility were unlikely to be the reason for hyperactivity of PhoQ-Ala_20_Pro. Instead, activation of the receptor was mediated by an aromatic or heterocyclic ring at this position, possibly due to confined mobility of TM1 or altered interactions with the second PhoQ protomer.

### Ala_20_Pro alters the impact of allosteric inhibitors of PhoQ

Since the TM1 helix is important for signal transduction [47], we asked how PhoQ-Ala_20_Pro responded to two known allosteric inhibitors, i.e., Mg^2+^ and MgrB. Adding magnesium to growth media at saturating concentrations suppressed *PphoP* promoter activity by ~35% in the wild type strain. For Δ*mgrB* and the PhoQ-Ala_20_Pro knock-in, a greater inhibition of ~50% was observed (Fig 5E). However, though *PphoP* activity was reduced to the level of the *phoQ* knockout in wild type and *ΔmgrB*, it remained ~2-fold higher in the PhoQ-Ala_20_Pro knock-in strain even at the highest magnesium concentration (Fig 5F). Since these experiments were performed in LB medium, which may have a basal concentration of Mg^2+^ ions, we also repeated these experiments in M9 (minimal medium) supplemented with 0.1, 1 and 10 mM Mg^2+^. Here too, increasing Mg^2+^ concentrations inhibited PphoP activity in a dose-dependent manner across all strains. At 0.1 mM Mg^2+^, loss of MgrB rather than activation by the Ala_20_Pro mutation led to higher activity at the lowest concentration tested, though at higher Mg^2+^ concentrations once again Ala_20_Pro showed greater activation (S1 Fig). Thus, the PhoQ-Ala_20_Pro receptor continued to be sensitive to magnesium; but this mutant could activate PhoP even at saturating concentrations of Mg^2+^. These data indicated that the Ala_20_Pro mutation altered the basal activity of PhoQ, possibly by inducing a conformational change that locked the kinase domains in an active conformation.

The second allosteric inhibitor of PhoQ, i.e., MgrB, binds in the transmembrane region. It has been suggested that displacement of MgrB by cationic peptides leads to receptor activation [48]. We wondered whether the Ala_20_Pro mutation also activated PhoQ by a similar mechanism, i.e., by preventing binding to MgrB. If true, deletion of *mgrB* from *E. coli* expressing PhoQ-Ala_20_Pro would have no impact on TMP resistance. On the other hand, if MgrB continued to inhibit PhoQ-Ala_20_Pro, loss of the repressor would further increase TMP resistance. Contrary to both expectations, MgrB-deficiency reduced colony formation on TMP-supplemented media for plasmid-borne as well as genomically encoded PhoQ-Ala_20_Pro (Fig 5G, S2 File). Fold change in TMP IC50 values confirmed negative epistasis (ε = −0.047, σε = 0.02) between PhoQ-Ala_20_Pro and *ΔmgrB* (Fig 5H). DHFR expression was also lower in the double mutant than in the PhoQ-Ala_20_Pro knock-in (Fig 5I).

Immunoblotting revealed that in the absence of MgrB, protein level of PhoQ-Ala_20_Pro was substantially lower than in its presence (Fig 5J). Wild type PhoQ protein expression was unaffected by MgrB. Interestingly, the level of the Ala_20_Trp mutant, which also resulted in TMP resistance, was unaffected by MgrB-deficiency (Fig 5J). In line with this observation, its resistant phenotype was also unaffected by loss of MgrB (S2 File). These results indicated that substitution of Ala_20_ to Pro led to destabilisation of the PhoQ protein and likely rendered it susceptible to proteolysis. MgrB stabilised the mutant protein, either by directly binding to it or through an indirect effect, leading to greater activation of PhoP and higher TMP resistance. Importantly, this also explained why PhoQ-Ala_20_Pro occurred as a stand-alone mutation rather than in combination with *mgrB* mutations in our evolution experiment.

### Gene regulatory effects of PhoQ pathway mutants depend on its activity level

In *E. coli*, 55 protein coding genes are known to be direct targets of PhoP [49]. Interestingly, some of the targets of PhoP are themselves regulators of gene expression, and in a previous study we showed that loss-of-function mutations in MgrB affect the primary and secondary targets of this pathway [30]*.* We, therefore, used RNA-sequencing to ask how activation of the PhoQP pathway by a naturally occurring polymorphism and mutations that arose under antibiotic selection affected gene expression in *E. coli*. For these analyses, in addition to PhoQ-Val_382_Gly (mildly activated PhoQP) and PhoQ-Ala_20_Pro (hyperactivated PhoQP) knock-in strains, we also included wild type, Δ*mgrB* (de-repressed PhoQP) and the *phoQ* knockout for comparison.

**Fig 6:**
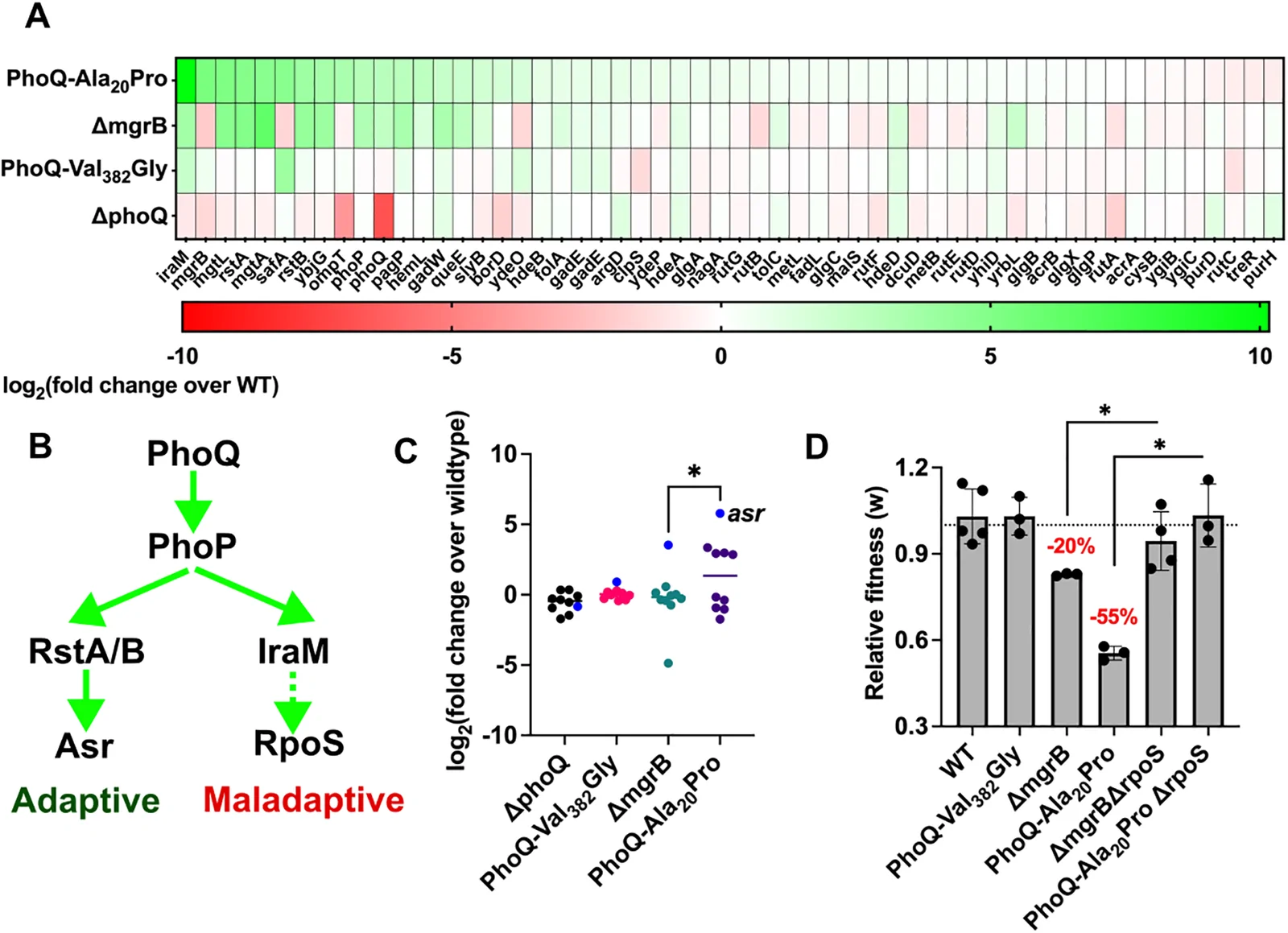
Deregulation of gene expression depends on activity level of PhoQ. **A:** Heatmap showing the expression levels of individual genes of the PhoP regulon on a green-red scale as indicated. Genes are arranged according to their deregulation in the PhoQ-Ala_20_Pro knock-in strain **B:** A schematic showing two pathways cross-activated by the PhoQ/PhoP two component system. Activation of the RstAB-Asr pathway is adaptive in TMP, while activation of IraM-RpoS is maladaptive resulting in a fitness cost. **C:** Expression level changes for RstA-regulated genes in the indicated strains. Individual points indicate genes of the RstA regulon, while the mean expression of the regulon is shown as a line. Asr gene expression scales with PhoQ activity and is highlighted in blue. Statistical significance was tested using a Welch’s *t* test, *p-value ≤ 0.05. **D:** Relative fitness (w) of indicated PhoQ pathway mutants and their *ΔrpoS* derivates measured using competition with *E. coli ΔlacZ* in drug-free LB medium. No change in fitness would result in a value of 1 which is indicated by a line. Mean ± SD from at least 3 replicates is plotted as bars. Individual data points are also shown. Magnitudes of fitness costs of *E. coli ΔmgrB* and PhoQ-Ala_20_Pro knock-in strains are expressed as percentages and indicated above the respective bars. Statistical significance was tested using a Welch’s *t* test, *p-value ≤ 0.05. The data underlying this Figure can be found in S4 File.

The natural variant allele PhoQ-Val_382_Gly led to almost no change in the expression of the PhoP-regulon (Fig 6A, S4 File). However, de-repression due to loss of MgrB or hyperactivation due to PhoQ-Ala_20_Pro resulted in pronounced up-regulation of several PhoP-target genes (Fig 6A, S4 File). Notably, though PhoQ-Ala_20_Pro resulted in greater up-regulation of the many genes than loss of MgrB, this was not reflected in all PhoP-targets (Fig 6A, S4 File). For instance, levels of *phoP* transcript itself were comparable between the de-repressed and hyperactive states (8.5-fold and 7.5-fold over wild type, respectively). These data suggested the different levels of active PhoQP translated to different expression levels for only a sub-set of the PhoP regulon.

Through its transcriptional targets, PhoP activates other regulatory pathways in *E. coli* (Fig 6D). One such pathway implicated in adaptation to TMP is the RstAB two-component system and its regulon, including the acid-resistance protein Asr (Fig 6B and 6C, S4 File) [30]. Asr expression was ~11-fold higher in the *ΔmgrB* strain, while it was up-regulated by ~51-fold in the PhoQ-Ala_20_Pro knock-in (Fig 6C). Interestingly, the most highly up-regulated PhoP-target gene in the PhoQ-Ala_20_Pro knock-in was *iraM* (Fig 6A and 6C, S4 File). Its expression was elevated ~1,000 fold in the PhoQ-Ala_20_Pro knock-in, while it was induced by ~12 fold in the *ΔmgrB* strain (Fig 6A, S4 File). We have shown earlier that IraM-dependent overproduction of RpoS-regulated genes imposes a fitness cost [30]. This fitness cost can be alleviated by deleting either *iraM* or *rpoS*. The massive overexpression of *iraM* in the PhoQ-Ala_20_Pro knock-in suggested that hyperactivation of PhoQ was even more costly than de-repression. In line with this result, the PhoQ-Ala_20_Pro knock-in had a lower relative fitness than the *ΔmgrB* strain in drug-free media, and deletion of *rpoS* completely eliminated this cost (Fig 6D). Importantly, PhoQ-Val_382_Gly led to only a mild induction of *iraM* (Fig 6A, S4 File) and no detectable cost to fitness (Fig 6D). Based on these results, we concluded that mutations altering PhoQ activity that emerged under TMP-selection led to deregulation of gene expression due to a combination of primary and secondary effects. On the other hand, a naturally occurring activating polymorphism only mildly perturbed the gene regulatory network of *E. coli* and was not detrimental for fitness in the absence of drug*.*

### Fitness cost limits high activity mutations in PhoQ during adaptation to TMP

**Fig 7:**
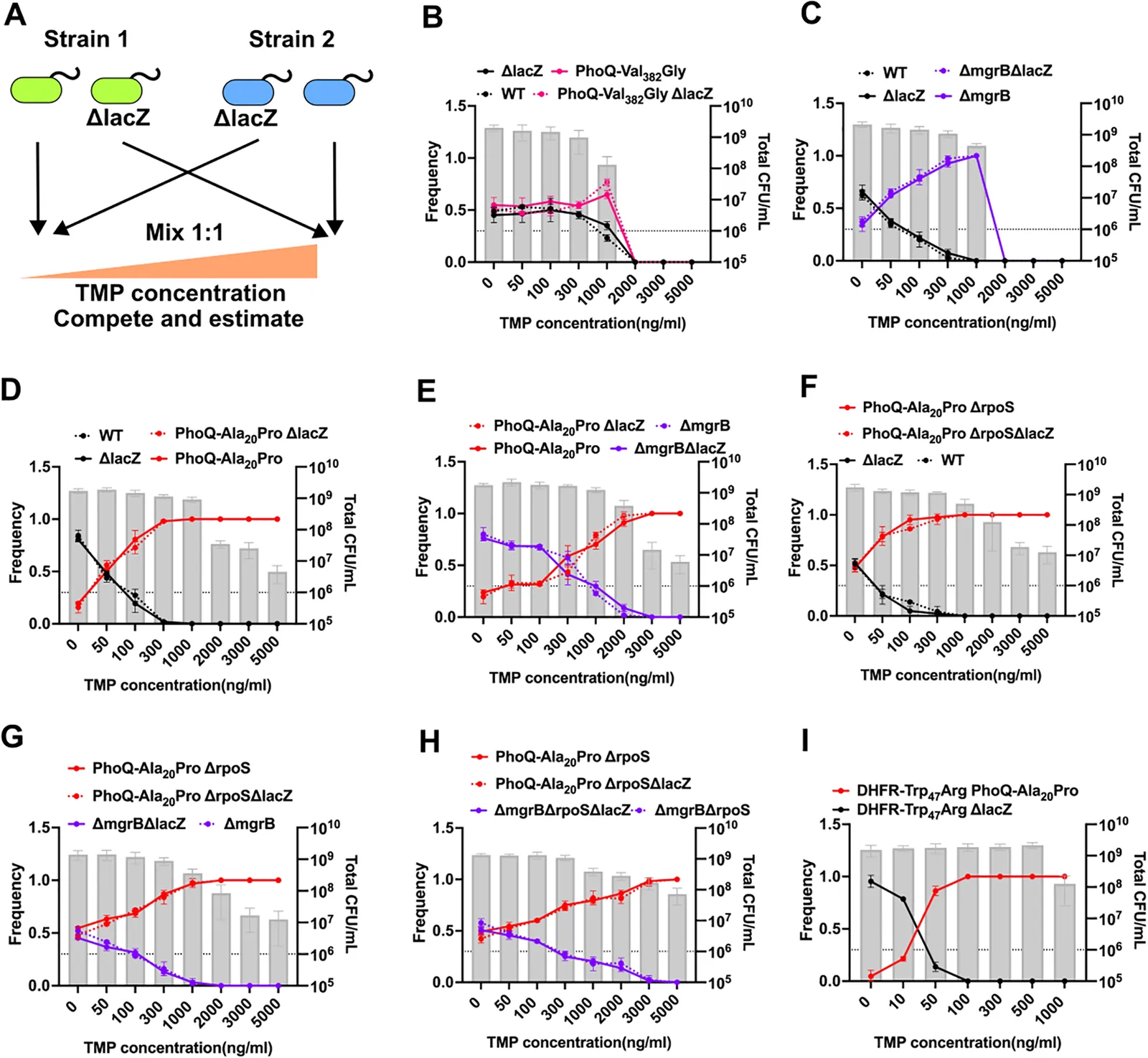
Selection of different PhoQ activity levels depends on TMP selection strength and fitness cost due to RpoS cross-activation. **A:** Schematic for pairwise competition between PhoQ pathway mutants performed across different trimethoprim concentrations to determine the relationship between TMP selection pressure and optimal PhoQ activity **B-H:** Results of pairwise competition between indicated strains with difference PhoQ activity levels. For each competition a pair of *lacZ*-expressing and *lacZ*-deficient strains were mixed at an initial ratio of 1:1 (i.e., frequency of 0.5 for each of the two strains in the mixture). Frequency of each competitor after competition (left Y-axis) was calculated. Total CFU/mL of the mixed cultures is plotted on the right Y-axis as bars. Limit of detection (~10^6^ CFU/mL) is shown as a dotted line. Mean ± SD from 3 replicates is shown. **I:** Competition between DHFR-Trp_47_Arg knock-in harbouring a *ΔlacZ* marker and TMP-adapted DHFR-Trp_47_Arg knock-in harbouring the PhoQ-Ala_20_Pro mutation (Line 7) at different TMP concentrations. Frequency of each competitor after competition (left Y-axis) was calculated. Total CFU/mL of the mixed culture is plotted on the right Y-axis as bars. Limit of detection (~10^6^ CFU/mL) is shown as a dotted line Mean ± SD from 3 replicates is plotted. The data underlying this Figure can be found in S5 File.

The above experiments demonstrated that for mutations altering PhoQ activity, TMP resistance-level scaled with fitness cost. This trend led us to ask how the optimal activity of the PhoQ-PhoP-DHFR pathway was set during evolution, i.e., what was the relationship between drug pressure and the most preferred activation state of PhoQ. First, we used pair-wise co-cultures to assess how TMP altered allele frequencies of mildly active (PhoQ-Val_382_Gly), derepressed (*ΔmgrB*) and hyperactive (PhoQ-Ala_20_Pro) mutants in competition with wild type. Competing strains were mixed at a 1:1 ratio (i.e., an initial frequency of 0.5) in drug-free or TMP-supplemented medium and changes in their frequency after a single growth cycle were assessed. Any deviation from a frequency of 0.5 indicated a preference for one of the two competing strains (Fig 7A).

In line with its mildly resistant phenotype and no detectable cost, PhoQ-Val_382_Gly knock-in and wild type remained equi-proportionate over much of the TMP concentration range. However, close to MIC_wt_ (~1 μg/mL) the PhoQ-Val_382_Gly knock-in was enriched, indicating that even though this polymorphism had a small impact on PhoQ activity, it provided sufficient benefit to be selected by TMP (Fig 7B). Yet, at TMP concentrations higher than MIC_wt_, both, wild type and the knock-in were driven to extinction, demonstrating a narrow selection window (Fig 7B). On the other hand, *ΔmgrB* and the PhoQ-Ala_20_Pro knock-in strain increased in frequency over a much wider range of TMP concentrations, though neither was preferred in the absence of antibiotic commensurate with their fitness costs (Fig 7C and 7D). Key differences between de-repressed and hyperactivated PhoQ were apparent. *E. coli ΔmgrB* was selected over wild type starting from the lowest concentration of antibiotic tested, i.e., 50 ng/mL (MIC_wt_/20), while the PhoQ-Ala_20_Pro knock-in required a higher drug concentration (100 ng/mL, MIC_wt_/10) to offset its larger fitness cost (Fig 7C and 7D). Furthermore, though the *ΔmgrB* strain did not grow beyond MIC_wt_, the PhoQ-Ala_20_Pro knock-in survived till the highest drug concentration tested.

Next, we performed pairwise competitions between *ΔmgrB* and the PhoQ-Ala_20_Pro knock-in. Interestingly, the PhoQ-Ala_20_Pro knock-in was enriched over *ΔmgrB* only at TMP concentrations approaching the MIC_wt_, i.e., at 1 μg/mL or above (Fig 7E). On the other hand, *ΔmgrB* out-competed the hyperactive mutant at all sub-MIC_wt_ concentrations including in the absence of antibiotic (Fig 7E). This observation indicated that at sub-MIC_wt_ pressures fitness cost was the main factor determining the preferred adaptive strategy, while at higher pressures resistance level primarily determined the outcome. To confirm this idea, we performed similar competition assays between the PhoQ-Ala_20_Pro knock-in harbouring a deletion in *rpoS* and *E. coli* wild type or *ΔmgrB*. Indeed, the cost-compensated PhoQ-Ala_20_Pro knock-in out-competed both the wild type and *ΔmgrB* strains across the entire range of TMP pressures (Fig 7F and 7G). When both, de-repressed and hyperactive PhoQ were cost-compensated, once again, the PhoQ-Ala_20_Pro knock-in was selected over much of the TMP concentration range tested, verifying our model (Fig 7H).

The above results cemented the impact of fitness cost due to cross-activation of RpoS in limiting the evolution of high activity mutations in the PhoQ. The repeated isolation of *mgrB* mutations and the almost complete absence of mutations in PhoQ during several independent laboratory evolution experiments performed here and in the past [27,29,50] resonated strongly with this result. However, it raised the question of why a hyperactivating mutation in PhoQ was so readily isolated in the DHFR-Trp_47_Arg knock-in. A possible explanation was that since this strain was hypersensitive to begin with, the presence of a pre-existing deleterious mutation would alter the selection dynamics of PhoQ-Ala_20_Pro. To test this, we allowed the DHFR-Trp_47_Arg knock-in and the TMP-adapted population harbouring PhoQ-Ala_20_Pro to compete at different drug pressures. Interestingly, the adapted population was enriched over its ancestor even at 50 ng/mL of TMP and almost completed out-competed its ancestor by 100 ng/mL of TMP (Fig 7I). Thus, the presence of a deleterious natural variant allele at the DHFR locus facilitated the selection of a hyperactive mutant of PhoQ despite its high cost. These results demonstrated that polymorphisms in resistance genes not only altered the evolvability of bacteria under drug pressure, but also which mutations were likely to be selected by antibiotics.

## Discussion

**Fig 8:**
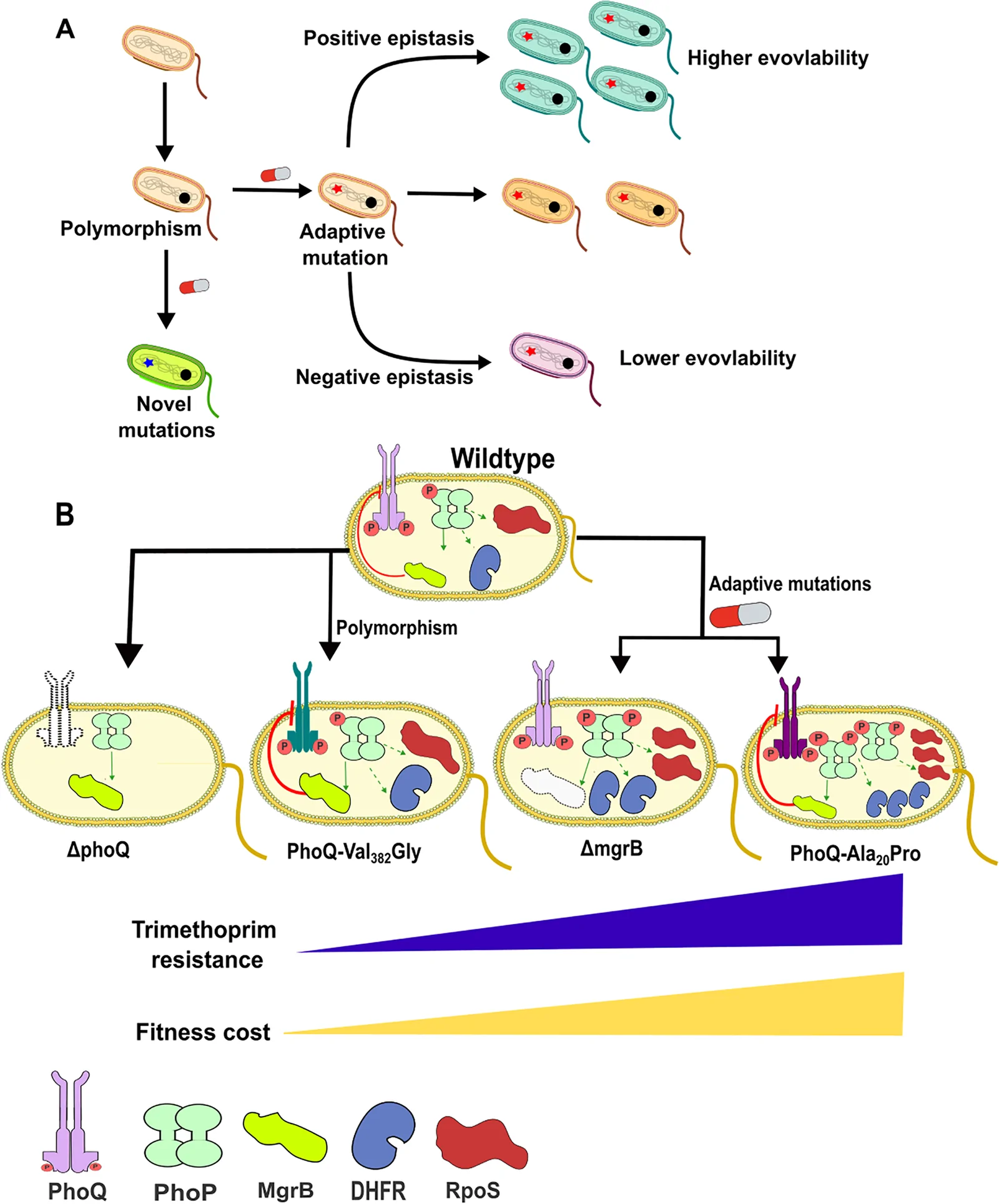
Proposed model for the contribution of polymorphisms towards evolvability of resistance, and the role of fitness cost of TMP resistance in determining the optimal level of PhoQ activity during evolution. **A:** Polymorphisms influence evolvability under drug pressure if they have epistatic interactions with adaptive mutations. Positive epistasis between pre-existing polymorphisms and adaptive mutations promotes the evolution of resistance, i.e., enhances evolvability of resistance. In contrast, negative epistasis diminishes evolvability. In addition, polymorphisms with negative epistasis can also drive adaptation to antibiotics through novel mutational paths. **B:** PhoQP signalling pathway across different mutants from the present study. The impact of PhoQ activity on trimethoprim resistance due to DHFR expression is correlated with fitness cost due to RpoS cross-activation. Genetic variation in the pathway is brought about by naturally occurring polymorphisms or drug-selected adaptive mutations. These genetic changes in the pathway activate signalling to different extents at the molecular level, thereby altering global transcriptome to different extents. Scaling of trimethoprim resistance level and fitness cost leads to changes in the concentration of antibiotic required to select for different levels of PhoQ/PhoP pathway activity. The presence of a hypersensitising allele of DHFR drove *E. coli* towards a high activity, high cost PhoQ mutant demonstrating how polymorphisms can change the mutational landscape of adaptation to antibiotics.

Genetic polymorphisms in bacterial two-component systems are associated with phenotypes such as stress tolerance and virulence. Despite mechanistic understanding of how polymorphisms alter bacterial physiology, their role in adaptation is not clear. In this study, we uncovered naturally occurring polymorphisms in the PhoQ-PhoP-DHFR pathway that modulate intrinsic susceptibility of *E. coli* to TMP, as well as evolvability under drug pressure. In the case of Val_382_Gly, an activating polymorphism in PhoQ, positive epistasis with an early adaptive mutation increased evolvability. On the other hand, the DHFR-Trp_47_Arg variant reduced evolvability by sensitising *E. coli* to TMP and negative epistasis with mutations in the drug target. Though opposing in their effects, these two results are linked by a common genetic mechanism, i.e., epistasis between pre-existing mutations and those that emerge during adaptation (Fig 8A). Indeed, epistasis is a strong force shaping adaptation and previous studies have highlighted its role in historical contingency during evolution [51], as well as propagation and amplification of small fitness effects over genetic/metabolic networks [52]. The present study revealed that epistatic interactions change evolutionary outcomes, both quantitatively and qualitatively. The quantitative impact was reflected in the frequency of replicate populations that evolved resistance during antibiotic challenge. The qualitative impact was seen as a change in the fitness landscape of adaptive mutations. Loss of *mgrB*, a first step mutation during the evolution of TMP resistance, is a low-level resistant mutant by itself [27]. But the pre-existence of PhoQ-Val_382_Gly substantially potentiated its resistance phenotype. Likewise, Trp_47_Arg in DHFR exacerbated the instability of resistance-conferring mutations, reducing their resistance level. On the other hand, though the high resistance phenotype of PhoQ-Ala_20_Pro was consistent regardless of the allele of DHFR, its selection as a spontaneous stand-alone mutation was facilitated by the Trp_47_Arg variant of DHFR.

Differing evolvability due to genetic variation provides a likely explanation for why certain strains of pathogenic bacteria are more prone to evolve multidrug resistance than others. A few examples from published literature support this idea. For instance, Ortiz and colleagues used extensive genomics analyses to identify “pre-resistance mutations” in some lineages of *Mycobacterium tuberculosis* [53]. These polymorphisms, though not themselves primary determinants of resistance were associated with a higher propensity for multidrug resistance, and could serve as predictors of the risk of resistance evolution. Similarly, Jangir and colleagues showed that the presence of polymorphisms in cell wall biosynthetic genes of pathogenic *E. coli* strains enhanced evolvability of resistance to colistin, a last resort antibiotic that targets the lipopolysaccharide layer in gram-negative bacteria [19]. We propose that understanding how pre-existing polymorphisms interact with adaptive mutations is an important direction to pursue for predicting risk of resistance evolution. Genome-wide association studies (GWAS) on antibiotic resistant pathogens frequently identify a number of variants linked with resistance [54]. However, most experimental validation pipelines focus on highest effect variants that are usually themselves determinants of resistance, while the role of others remain uncertain. As an example, the study by Diaz Caballero and colleagues identified polymorphisms associated with beta-lactam resistance in *Burkholderia multivorans* [55]. In addition to mutations in AmpD, a gene that is known to be associated with beta-lactam resistance across bacteria, the authors identified a large number of variants at other loci that were statistically highly likely to be involved in resistance. Strikingly, phosphorelay mechanisms including two-component systems were enriched, though their precise role was unexplored [55]. We propose that validation pipelines must also take into account effects on evolvability of resistance, which in the case of regulatory proteins may be profoundly important as demonstrated by our work.

The PhoQ-PhoP two-component system, originally discovered in Mg^2+^ homeostasis and acid adaptation, has emerged as a major regulator of antibiotic resistance in Enterobacteria. In addition to modulating TMP resistance in *E. coli*, its role in colistin resistance in *Klebsiella pneumonia* is well studied [56]. Additionally, an association with carbapenem resistance in *Enterobacter* sp. has been reported as well [57]. The wide range of phenotypes are ascribed to several downstream effectors creating a cascade of gene regulatory changes. Some of these effectors are direct targets, while others are regulated by targets of PhoP [43]. A major downstream pathway that we analysed in the present work was the stress responsive RpoS regulon, which manifests in a fitness cost [30]. Importantly, since cost of PhoQ activation scaled with benefit (i.e., TMP resistance), it capped the evolution of PhoQ activity during adaptation and prevented the fixation of high activity mutations such as PhoQ-Ala_20_Pro during antibiotic challenge (Fig 8B). Indeed, pairwise competition experiments showed that unless drug pressure was high (i.e., > MIC_wt_), moderate activation of PhoQ, i.e., by de-repression, was the “fitter” strategy. The relationship between cross-talk and evolution of signalling pathways has been an area of active investigation for more than a decade. For bacterial signalling, particularly two-component systems, cross-talk has been viewed as undesirable. Indeed, many studies have investigated the insulation of signalling pathways as an evolutionary mechanism to improve signalling fidelity and organismal fitness [58–60]. In contradiction, instances of cross-activating transcriptional programs triggered by two-component systems are known. For instance, in *E. coli* small ‘connector’ proteins synthesise the impact of different two-component systems on gene expression [61–63]. Similarly, more than one two-component signalling system activates the stress-responsive sigma factor, RpoS [64]. The results presented in the present study provide an explanation for this apparent dichotomy. Our work shows that when signalling pathways evolve towards higher activity levels, cost due to cross-activation is likely only relevant above a threshold of activity (Fig 8B). The PhoQ-Val_382_Gly mutation, for instance, activated PhoP to provide an advantage in TMP without detectable cost. Signalling pathways may, thus, have some ‘wiggle room’ for tuning their activity. Ultimately, the strength of selection for higher activity would determine whether a signalling pathway overcomes the cost of cross-activation during its evolution.

Curiously, the impact of different activating mutations in the PhoQ-PhoP pathway did not scale with active PhoP for all target genes. We noticed two distinct patterns in terms of the responsiveness of gene promoters to active PhoP levels. First class of promoters had low sensitivity, i.e., they were affected only at moderate and high PhoQ activity, but were unaffected by mild activation or inactivation of the signalling pathway. The *phoPQ* operon itself also fell into this category. Importantly, these promoters also showed saturation, i.e., their expression level was comparable between de-repressed and hyperactivated states of PhoQ (Fig 6A). The second type of promoter was highly sensitive to PhoQ activity. Indeed, the *iraM* promoter best exemplified this category as its expression level was altered upon loss and gain of PhoQ activity. Interestingly, despite being highly sensitive, expression of *iraM* did not saturate with PhoQ activity, i.e., de-repression and hyperactivation of PhoQ were clearly reflected in the levels of *iraM* (Fig 6A). When we analysed the promoters of *iraM* and *phoP* as representative of the two types, we noticed that both promoters had two PhoP binding sites each. For *iraM*, one of the sites had a greater similarity to the PhoP-binding consensus sequence, indicating high affinity, while the other was likely to be a low affinity site [64,65]. On the other hand, both PhoP sites in the *phoP* promoter were expected to be high affinity sites [66,67]. Further, both *iraM* promoter sites are predicted to be activating, while one of the PhoP-binding sites in the *phoP* promoter is expected to be repressive. Thus, differences in promoter architecture likely contributed to the differences in responsiveness to active PhoP. Though, these speculations require mechanistic dissection and experimental validation, their implications are significant, as differential impact of activation of a signalling pathway on its targets would mean that not all targets contribute equally towards the fitness effects of activating mutations. By extension, the evolution of a signalling pathway may be driven primarily by only a subset of its regulon and which subset drives evolution may in turn depend on the environment. We propose that the PhoQ-PhoP signalling pathway evolving under trimethoprim pressure may prove to be a good model to test these ideas.

In summary, the work presented here identifies the contribution of pre-existing polymorphisms in a pathway associated with TMP resistance in determining evolvability of resistance. We also uncover how benefit and cost shape the evolution of a signalling pathway under selection. These findings establish the PhoQ-PhoP-DHFR pathway as a paradigm for studying the evolution of a drug resistance-associated pathway at the molecular and organismal levels

## Materials and methods

### Bacterial strains and culture conditions

Luria Bertani broth (LB) or agar (LA) was used to culture *E. coli* strains. All strains were cultures at 37 °C and broth cultures were grown with shaking at 180 rpm. Growth media were supplemented with different concentrations of trimethoprim (TMP) or magnesium sulphate as needed. Chloramphenicol (Cmp, 30 μg/mL), ampicillin (Amp, 100 μg/mL), kanamycin (Kan, 30 μg/mL), gentamycin (Gent, 45 μg/mL) and streptomycin (Strp, 45 μg/mL) were added to the media for the selection of transformants and knockouts as needed. List of strains and plasmids used in this study are provided in Tables 1 and 2, respectively.

**Table 1:** Strains used in this study.

Sr. no.	Strain name	Description	Source
1	*E. coli* K-12 MG1655	Wild-type *E. coli* used as ancestor for evolution experiments and as reference strains for phenotypic and genotypic comparisons.	Available in the laboratory
2	*E. coli* DH10B	*E. coli* strain used for standard cloning and genetic manipulation of plasmids	Available in the laboratory
3	*E. coli* CC118	*E. coli* strain for propagation and manipulation of pSEVA612S plasmid which contains an R6K origin of replication.	Kind gift from Prof. Gad Frankel, Imperial College, London, U.K.
4	*E. coli* pRK2013 Helper strain	*E. coli* Helper Strain for triparental conjugation	Kind gift from Prof. Gad Frankel, Imperial College, London, U.K.
5	*E. coli ΔphoQ*	Knockout of the *phoQ* gene (*ΔphoQ*::*Kan*^*R*^)	[27,30]
6	*E. coli ΔmgrB*	Knockout of the *mgrB* gene (*ΔmgrB*::*Kan*^*R*^)	[27,30]
7	*E. coli ΔphoQ::FRT*	Knockout of the *phoQ* gene, with the Kanamycin resistance cassette removed	This study
8	*E. coli ΔphoQ::*PhoQ-Val_382_Gly	Scarless genomic knock-in of the *phoQ* gene harbouring Val_382_Gly mutation	This study
9	*E. coli ΔphoQ::*PhoQ-Val_382_Gly *ΔmgrB*	Knockout of *mgrB* (*mgrB*::Kan^R^) in *E. coli ΔphoQ::*PhoQVal_382_Gly knock-in strain	This study
10	*E. coli* DHFR-Trp_47_Arg	Scarless genomic knock-in of the *folA* gene harbouring Trp_47_Arg mutation	This study
11	*E. coli ΔphoQ::*PhoQ-Ala_20_Pro	Scarless genomic knock-in of the *phoQ* gene harbouring Ala_20_Pro mutation	This study
12	*E. coli ΔphoQ::*PhoQ-Ala_20_Pro *ΔmgrB*	Knockout of *mgrB* (*ΔmgrB*::Kan^R^) in *E. coli ΔphoQ::*PhoQ-Ala_20_Pro knock-in strain	This study
13	*E. coli ΔmgrBΔrpoS*	Double knockout of *mgrB* and *rpoS* genes (Δ*mgrB::FRT rpoS::Kan*^*R*^)	[27,30]
14	*E. coli ΔphoQ::*PhoQ-Ala_20_Pro *ΔrpoS*	Knockout of *rpoS* (rpoS::Kan^R^) in *E. coli ΔphoQ::*PhoQAla_20_Pro knock-in strain	This study
15	*E. coli ΔlacZ*	Knockout of the *lacZ* gene (*ΔlacZ::cat*)	[39]
16	*E. coli ΔmgrB ΔlacZ*	Double knockout of *mgrB* and *lacZ* genes (*mgrB::Kan*^*R*^ *lacZ::cat*)	This study
17	*E. coli ΔphoQ::*PhoQ-Val_382_Gly *ΔlacZ*	Knockout of *lacZ* (*ΔlacZ::cat*) in *E. coli ΔphoQ::*PhoQ-Val_382_Gly knock-in strain	This study
18	*E. coli ΔphoQ::*PhoQ-Ala_20_Pro *ΔlacZ*	Knockout of *lacZ* (*ΔlacZ::cat*) in *E. coli ΔphoQ::*PhoQ-Ala_20_Pro knock-in strain	This study
19	*E. coli ΔphoQ::*PhoQ-Ala_20_Pro*ΔrpoS ΔlacZ*	Double knockout of *rpoS* and *lacZ* (Δ*rpoS::Kan*^*R*^ *lacZ::cat*) in *E. coli ΔphoQ::*PhoQ-Ala_20_Pro knock-in strain	This study
20	*E. coli ΔmgrB ΔrpoS ΔlacZ*	Triple knockout of *mgrB*, *rpoS,* and *lacZ (ΔmgrB::FRT rpoS::Kan ΔlacZ::cat)*	This study
21	*E. coli* DHFR-Trp_47_Arg Line 1, Line 2, Line 5, Line 7	Trimethoprim resistant derivatives of *E. coli* DHFR-Trp_47_Arg obtained by passing the strain for 12 growth cycles in 500 ng/mL of trimethoprim	This study

**Table 2:** Plasmids used in this study.

Sr. no.	Plasmid	Description	Source
1	pCA24N-PhoQ	pCA24N containing a wild-type copy of the *phoQ* gene with an N-terminal hexa-Histidine tag under the T5-lac promoter, CMP^R^. The pCA24N plasmid is a high-copy plasmid (200–300 copies/cell) with a pMB1 origin.	ASKA Library [68]
2	pCA24N-PhoQ^L131I^	Plasmid pCA24N-PhoQ containing Leu_131_Ile mutation in PhoQ, CMP^R^	This study
3	pCA24N-PhoQ^V163I^	Plasmid pCA24N-PhoQ containing Val_163_Ile mutation in PhoQ, CMP^R^	This study
4	pCA24N-PhoQ^E222K^	Plasmid pCA24N-PhoQ containing Glu_222_Lys mutation in PhoQ, CMP^R^	This study
5	pCA24N-PhoQ^Q317L^	Plasmid pCA24N-PhoQ containing Gln_317_Leu mutation in PhoQ, CMP^R^	This study
6	pCA24N-PhoQ^F372V^	Plasmid pCA24N-PhoQ containing Phe_372_Val mutation in PhoQ, CMP^R^	This study
7	pCA24N-PhoQ^V382G^	Plasmid pCA24N-PhoQ containing Val_382_Gly mutation in PhoQ, CMP^R^	This study
8	pCA24N-PhoQ^E464D^	Plasmid pCA24N-PhoQ containing Glu_464_Asp mutation in PhoQ, CMP^R^	This study
9	pCA24N-PhoQ^D485G^	Plasmid pCA24N-PhoQ containing Asp_485_Gly mutation in PhoQ, CMP^R^	This study
10	pCA24N-PhoQ^V382P^	Plasmid pCA24N-PhoQ containing Val_382_Pro mutation in PhoQ, CMP^R^	This study
11	pCA24N-PhoQ^V382D^	Plasmid pCA24N-PhoQ containing Val_382_Asp mutation in PhoQ, CMP^R^	This study
12	pCA24N-PhoQ^V382R^	Plasmid pCA24N-PhoQ containing Val_382_Arg mutation in PhoQ, CMP^R^	This study
13	pCA24N-PhoQ^V382W^	Plasmid pCA24N-PhoQ containing Val_382_Trp mutation in PhoQ, CMP^R^	This study
14	pCA24N-PhoQ^A20P^	Plasmid pCA24N-PhoQ containing Ala_20_Pro mutation in PhoQ, CMP^R^	This study
15	pCA24N-PhoQ^A20W^	Plasmid pCA24N-PhoQ containing Ala_20_Trp mutation in PhoQ, CMP^R^	This study
16	pCA24N-PhoQ^A20F^	Plasmid pCA24N-PhoQ containing Ala_20_Phe mutation in PhoQ, CMP^R^	This study
17	pCA24N-PhoQ^A20K^	Plasmid pCA24N-PhoQ containing Ala_20_Lys mutation in PhoQ, CMP^R^	This study
18	pCA24N-PhoQ^A20E^	Plasmid pCA24N-PhoQ containing Ala_20_Glu mutation in PhoQ, CMP^R^	This study
19	pCA24N-PhoQ^A20G^	Plasmid pCA24N-PhoQ containing Ala_20_Gly mutation in PhoQ, CMP^R^	This study
20	pCA24N-PhoQ^A20I^	Plasmid pCA24N-PhoQ containing Ala_20_Ile mutation in PhoQ, CMP^R^	This study
21	pCA24N-PhoQ^A20V^	Plasmid pCA24N-PhoQ containing Ala_20_Val mutation in PhoQ, CMP^R^	This study
22	pPRO-folA	Wild type *folA* gene cloned into the pPROEx-HtB expression plasmid, Amp^R^	[39]
23	pPRO-folA^W47R^	Plasmid pPRO-folA containing Trp_47_Arg mutation in FolA, Amp^R^	This study
24	pPRO-folA^P105A^	Plasmid pPRO-folA containing Pro_105_Ala mutation in FolA, Amp^R^	This study
25	pPRO-folA^P21L^	Plasmid pPRO-folA containing Pro_21_Leu mutation in FolA, Amp^R^	[38,39]
27	pPRO-folA^P21L W47R^	Plasmid pPRO-folA containing Trp_47_Arg and Pro_21_Leu mutations in FolA, Amp^R^	This study
28	pPRO-folA^P21L-P105A^	Plasmid pPRO-folA containing Pro_105_Ala and Pro_21_Leu mutations in FolA, Amp^R^	This study
29	pPRO-folA^W30R^	Plasmid pPRO-folA containing Trp_30_Arg mutation in FolA, Amp^R^	[38,39]
30	pPRO-folA^W30R-W47R^	Plasmid pPRO-folA containing Trp_47_Arg and Trp_30_Arg mutations in FolA, Amp^R^	This study
31	pPRO-folA^W30R-P105A^	Plasmid pPRO-folA containing Pro_105_Ala and Trp_30_Arg mutations in FolA, Amp^R^	This study
32	pPRO-folA^F153A^	Plasmid pPRO-folA containing Phe_153_Ala mutation in FolA, Amp^R^	[38,39]
33	pPRO-folA^F153A-W47R^	Plasmid pPRO-folA containing Trp_47_Arg and Phe_153_Ala mutations in FolA, Amp^R^	This study
34	pPRO-folA^F153A-P105A^	Plasmid pPRO-folA containing Pro_105_Ala and Phe_153_Ala mutations in FolA, Amp^R^	This study
35	pPRO-folA^I94L^	Plasmid pPRO-folA containing Ile_94_Leu mutation in FolA, Amp^R^	[38,39]
36	pPRO-folA^I94L-W47R^	Plasmid pPRO-folA containing Trp_47_Arg and Ile_94_Leu mutations in FolA, Amp^R^	This study
37	pPRO-folA^I94L-P105A^	Plasmid pPRO-folA containing Pro_105_Ala and Ile_94_Leu mutations in FolA, Amp^R^	This study
38	pUA66-*P*_*phoP*_-GFP	Plasmid containing *phoP* promoter driving expression of GFP, Kan^R^	[69]
39	pUA66-*P*_*folA*_-GFP	Plasmid containing *folA* promoter driving expression of GFP, Kan^R^	[69]
40	pSEVA612S-GFP	pSEVA612S plasmid encoding GFP and harbouring an R6K origin of replication, Gent^R^	Kind gift from Prof. Gad Frankel, Imperial College, London, U.K.
41	pSEVA612S-*folA*^W47R^	pSEVA612S containing *folA*^*W47R*^ gene and *folA* homology regions, Gent^R^	This study
42	pSEVA612S-PhoQ^V382G^	pSEVA612S containing *phoQ*^*V382G*^ gene and *phoQ* homology regions, Gent^R^	This study
43	pSEVA612S-PhoQ^A20P^	pSEVA612S containing *phoQ*^*A20P*^ gene and *phoQ* homology regions, Gent^R^	This study
44	pACBSR	Temperature sensitive plasmid expressing I-*Sce*I endonuclease and Lambda recombinase system under arabinose inducible promoter, Strp^R^	Kind gift from Prof. Gad Frankel, Imperial College, London, U.K.

### Identification of naturally occurring polymorphisms in PhoQ, PhoP, and DHFR

To identify polymorphisms in proteins of interest, genome sequences available through the NCTC3000 database were mined [31]. Sample accession number of sequenced strains from NCTC3000 were acquired from the European Nucleotide Archive and the corresponding genome sequence was obtained from the Genbank. Amino acid sequences for the proteins of interest from each of the genomes were identified by looking for the appropriate annotation. Multiple sequence alignment was performed for PhoQ, PhoP, DHFR, BaeS, BasS, CpxA, and EnvZ proteins using Clustal W in Mega-X [70]. Protein sequences of each of these genes from *E. coli* K-12 MG1655 (U00096.3) were used as reference. Any deviation from the reference was recorded as a naturally occurring polymorphism. For each polymorphism, the frequency of occurrence was calculated as the ratio of the number of sequences harbouring the sequence variant and total number of sequences analysed.

For PhoP, PhoQ, and other sensor kinases domain boundaries were identified using the Interpro database [71]. Protein stability (ΔΔG values) for mutant PhoQ, PhoP, and DHFR was predicted using I-Mutant2.0 [37]. Values of ΔΔG between −1 and 1 kcal/mol were considered to not affect the stability of the protein, while any negative value below −1 was classified as destabilising.

### Site-directed mutagenesis of PhoQ and DHFR

PCR-based site-directed mutagenesis was used to introduce mutations into plasmid-borne *phoQ* and *folA* genes using a protocol described in Shenoy and Visweswariah, 2003 [72]. For the generation of PhoQ mutants, pCA24N-phoQ was used as a template, while for DHFR mutants, pPRO-folA was used as template. All mutagenic primers are listed in the S6 File. Amplified PCR product was digested overnight using *Dpn*I enzyme to remove the wild-type template. Digested product was then transformed into *E. coli* DH10B competent cells and selected on antibiotic-containing LA plates. Mutant plasmids were isolated from transformants using Alkali Lysis method, screened using restriction enzymes and confirmed by Sanger sequencing (Barcode Biosciences, India). For double mutants of DHFR, appropriate single mutants were used as template for the mutagenesis PCR.

### Measurement of trimethoprim resistance

#### Method 1: Colony formation assay.

Colony formation efficiency on trimethoprim-supplemented LA was performed as described in Balachandran and colleagues, 2025 [50]. Overnight-grown cultures of appropriate strains were serially diluted in LB using a 10-fold dilution series. Each dilution (10 μL) was spotted on LA plates containing different concentrations of trimethoprim. The trimethoprim concentrations for most assays ranged from 100 ng/mL to 6.4 µg/mL. Plates were incubated overnight at 37 °C, and the colony-forming units (CFU/mL) were determined. Log(CFU/mL) was used to compare growth across strains.

#### Method 2: Determination of Inhibitory concentration-50 (IC50).

The IC50 of trimethoprim from *E. coli* strains was determined using a Broth Microdilution Assay, as described in Vinchhi and Yelpure and colleagues, 2023 [30]. Briefly, glycerol stocks were revived in 3 mL of LB broth. Trimethoprim was serially diluted across the wells of a 96-well plate to obtain a 2-fold dilution series and 1% culture was inoculated. Final concentrations of trimethoprim ranged from 1 mg/mL to 0.47 ng/mL. The plates were incubated overnight at 37 °C, and optical density was measured at 600 nm using a microplate reader (Ensight, Perkin Elmer). Using GraphPad Prism software, the IC50 was determined by fitting the data to a nonlinear regression log-inhibitor versus response curve (GraphPad Prism, version 9.1.3) as described earlier [30,50]. The fold change in IC50 relative to wild type was calculated wherever necessary.

### Generation of scarless genomic knock-in strains of PhoQ and DHFR

Genomic knock-in strains were generated for PhoQ-Val_382_Gly, DHFR-Trp_47_Arg, and PhoQ-Ala_20_Pro using Triparental Conjugation based on the method adapted from Low and colleagues, 2022 [73]. For PhoQ-Val_382_Gly and PhoQ-Ala_20_Pro, the *phoQ* gene, together with flanking regions of homology, was cloned in the pSEVA612S plasmid using a three-part Gibson assembly. Primers used for generating these clones are listed in S6 File. Part 1 was amplified using *E. coli* wildtype genomic DNA as template and *PhoQ homology forward* and the *PhoQ mutagenic forward primers*. For part 2, *PhoQ homology reverse* and *PhoQ mutagenic reverse* primers were used. The third part was the pSEVA612S plasmid linearised using the *I-SceI primer* and pSEVA612S-GFP plasmid was used as template. The PCR products were *Dpn*I-digested overnight. The three parts were combined in a 3:1 insert:vector molar ratio using a Gibson Assembly Master Mix (New England Biolabs, U.S.A.) at 50 °C for 30 min. The mixture was then transformed into *E. coli* CC118 competent cells and plated on gentamycin-containing LA plates. GFP-negative colonies were screened using restriction digestion and positive clones were confirmed by Sanger sequencing (Genematrix LLP, Pune, India). To integrate the mutation into the *E. coli* genome, a triparental conjugation was performed. *E. coli* CC118 containing pSEVA-phoQ plasmid with appropriate mutation was used as the donor. The recipient strain *E. coli ΔphoQ::Kan*^*R*^ was transformed with the pACBSR plasmid, which contained a streptomycin-selectable marker gene and arabinose-inducible I-*Sce*I enzyme. *E. coli* pRK2013 helper strain was used for conjugation of pSEVA612S-phoQ plasmids from the donor to the recipient. For conjugation, overnight culture (20 µL) of the donor strain was spotted on LA and dried completely. Next, overnight culture (20 µL) of the helper stain was spotted over the donor strain, dried completely and incubated for 2 hours at 37 °C. Subsequently, 40 μL of the recipient was overlaid, dried and incubated for 4 hours at 37 °C. The growth obtained was scraped off the agar plate using a sterile nichrome loop, resuspended in 100 µL of fresh LB medium, spread on LA containing gentamycin and streptomycin and incubated at 37 °C overnight to select for single crossover merodiploid colonies. Three randomly selected colonies were inoculated into 3 mL LB, and I-*Sce*I enzyme and Lambda Red Recombinase present on the pACBSR plasmid were induced using 0.2% L-Arabinose. After 2 hours and 6 hours of induction at 37 °C a loopful of culture was streaked-out on LA plates and incubated overnight at 37 °C. Colonies from the LA plates were patched on kanamycin-containing plates, and those that had lost kanamycin resistance were selected*.* Scarless knock-ins were screened using genomic PCR and mutation-specific restriction digestion. The presence of the mutations was further confirmed using Sanger sequencing of PCR-amplified *phoQ* gene. Confirmed knock-ins were passaged on an LA plate for 5 days to cure the pACBSR plasmid from the cell and streptomycin-sensitive knock-ins were used for all experiments.

A similar procedure was followed for the generation of the DHFR-Trp_47_Arg knock-in. For the triparental conjugation, barring the recipient strain, all other steps were the same. The recipient used to generate the DHFR-Trp_47_Arg knock-in was *E. coli* K-12 MG1655 harbouring the pACBSR plasmid.

### Measuring phoP and folA promoter activity

Promoter activity for *phoP* and *folA* was measured using a GFP-reporter plasmid. Promoter reporter constructs were acquired from the *E. coli* promoter library (Dharmacon; P*phoP*- PEC3876-98154831, P*folA*-PEC3876-98155011). Strains harbouring the reporter plasmids were grown in 3 mL LB in the absence or presence of different concentrations of magnesium sulphate as needed. Overnight-grown cultures were diluted 1:1 in sterile LB, and 200 µL of the diluted cultures were used to measure GFP fluorescence (excitation-485 nm, emission-519 nm) and optical density (OD600). GFP fluorescence was normalised to OD600 to compare across strains. For testing the effect of magnesium, promoter activity in its presence was normalised to activity in the absence of magnesium.

### Immunoblotting for DHFR and PhoQ

DHFR protein levels in different strains were estimated using immunoblotting with anti-DHFR polyclonal IgG as described earlier [27,38]. For hexa-Histidine tagged PhoQ, anti-6xHis monoclonal IgG (MA1135, ThermoFisher, U.S.A.) was used. For DHFR, 5 µg of total cellular lysate protein was electrophoresed on a 15% SDS-PAGE gel and then electroblotted onto a PVDF membrane. For PhoQ, lysates were electrophoresed on a 12% SDS-PAGE gel. Membranes were blocked using 5% BSA for 1 hour at room temperature and incubated in primary antibody (100 ng/mL of anti-DHFR polyclonal IgG; 1 µg/mL anti-6xHis monoclonal IgG) at 4 °C overnight. HRP-linked anti-rabbit IgG (1:20,000) for DHFR and HRP-linked anti-mouse IgG (1:4000) for PhoQ were used as secondary antibody. Chemiluminescence was used to detect protein bands. FtsZ protein levels were used as a loading control. Anti-FtsZ polyclonal antiserum was used at a concentration of 1:50,000. Band intensities were quantitated using ImageJ and compared as required.

### Gene deletion in *E. coli* strains

All gene knockouts were generated using P1 transduction. P1 lysates were raised on appropriate knockout strain from the Keio Collection [74] carrying the Kanamycin-resistance marker and transduced into the appropriate host strain of *E. coli*. Kanamycin-resistant colonies were selected and knockouts were confirmed by PCR using gene specific primers. To generate an unmarked knockout, the kanamycin resistance marker was excised from the knockout strain using the pCP20 plasmid containing FLP recombinase, as described by Baba and colleagues, 2006 [74].

### Determination of evolvability for PhoQ-Val_382_Gly and DHFR-Trp_47_Arg knock-in strains

The difference in spontaneous resistance acquisition frequency across strains was assessed using an antibiotic challenge assay. Twenty-four replicate populations of appropriate genotype were inoculated in LB containing 100 ng/ml (0.1xMIC_wt_), 1,000 ng/mL(1xMIC_wt_), 2,000 ng/mL (2xMIC_wt)_ and 4,000 ng/mL (4xMIC_wt_) of trimethoprim in 96-well plates. The plates were incubated at 37 °C for 20–24 hours, which represented 1 growth cycle. At the end of a growth cycle, 1% of culture from each well was transferred to fresh LB containing trimethoprim. Antibiotic challenge was performed for 5 days, i.e., over 5 growth cycles. At the end of each growth cycle, optical density (OD600) was measured. OD600 > 0.1 was recorded as growth, while OD600 < 0.1 was recorded as no growth.

### Strain competitions and relative fitness

Relative fitness (w) was calculated using direct competition between test and reference strains that were distinguished by the presence or absence of *ΔlacZ::Cat* marker. The marker was swapped between strains for all experiments to establish its neutrality. Reference and test strains were grown as monocultures overnight in LB. They were then diluted 1:100 and combined in a 1:1 ratio in 3 mL of LB with or without appropriate concentration of trimethoprim. The initial CFU/mL for test and reference strains was assessed by serially diluting the mixed culture and plating on LA containing IPTG (1 mM) and X-Gal (50 µg/mL). The strains were allowed to compete for 24 hours at 37 °C with shaking at 180 rpm, after which CFU/mL was measured again. Relative fitness was calculated using the formula *w* = ln(T_f_/T_i_)/ln(R_f_/R_i_), where T_f_ and R_f_ were CFU/mL at the final time point, and T_i_ and R_i_ were CFU/mL at the initial time point for test and reference strains, respectively.

For pairwise competitions across different trimethoprim concentrations, similar co-cultures were set up. The frequency of each strain in the competition was measured by dividing the CFU/mL of the test strain by total CFU/mL.

### Adaptive laboratory evolution of trimethoprim resistance and genome sequencing

For laboratory evolution of trimethoprim resistance 15 replicates of DHFR-Trp_47_Arg knock-in and *E. coli* wild type were grown in the wells of a 96-well plate in LB supplemented with 500 ng/mL of trimethoprim for 12 growth cycles. Replicates were derived from independent cultures that originated from three different colonies of each genotype. Furthermore, evolution was performed in 3 sets, each consisting of 5 replicates, to prevent cross-contamination. Retrospective genome sequencing analyses was used to confirm that replicates within each set were indeed independent of each other, i.e., did not show identical mutations. Each growth cycle was for 24 hours after which 10% of the culture was passaged into fresh media. At the end of 12 passages, glycerol stocks were prepared for surviving lineages and stored at −80 °C.

For genome sequencing, frozen stocks of the surviving lineages were revived in 3 mL LB and grown till saturation. Genomic DNA was extracted using the phenol:choloroform:isoamyl alcohol and cleaned-up using spin columns. Paired-end, whole-genome Next Generation sequencing was performed on a MiSeq system (Illumina, USA) with read lengths of 150−200 bps. Library preparation and sequencing services were provided by Eurofins, India. Mutations were identified using Breseq pipeline [75]. The genome of *E. coli* K-12 MG1655 (Genbank:U00096.3) was used as reference. Raw sequencing reads are available in Genbank (PRJNA1398517).

### Transcriptome analysis using RNA sequencing

For RNA-sequencing, 1% of the overnight-grown cultures of appropriate strains were inoculated into 3 mL LB broth and incubated at 37 °C for 3 hours with shaking at 180 rpm. Three independent biological replicates were grown in parallel for these analyses. Cultures were centrifuged and pellets were resuspended in 1 mL of RNAlater (Invitrogen, U.S.A). Total RNA was extracted using Trizol, and the quality of the extracted RNA was estimated using TapeStation (Agilent). Depletion of rRNA was performed using the Ribozero kit/ NEBNext rRNA depletion kit. Following library preparation, total RNA-sequencing was performed on Illumina Miseq platform with a 150 bp read length. Processed reads were aligned to the reference genome (NC_00096.3/NC_0000913.3) using STAR(v 2.7.10a) and Strand NGS v4.1. Abundance estimation was done for counting reads corresponding to genes, promoter regions, and genomic bins using featureCounts (1.34.0). RNA extraction, ribodepletion, library preparation, sequencing and preliminary analysis were performed by Eurofins Genomics, India and Strand Lifesciences, India. Gene names were inferred from the gene ID for *E. coli* using the Ecocyc database [76]. PhoP-regulated genes were identified RegulonDB [49]. Differential gene expression analysis was performed using DESeq2 and EdgeR. All strains were compared to the wild type to obtain the Log_2_(fold change). Statistical significance was tested using the Benjamini–Hochberg test. A false discovery rate (FDR) cut-off of 0.05 was used to identify statistically significant gene expression changes. Raw sequencing reads are available in Genbank (PRJNA1399355).

### Statistical analyses

#### Testing for difference in evolvability across strains.

Cox proportional hazards model was used to assess differences in evolvability across strains in a drug-challenge assay. Analysis of the model and prediction of hazard ratio was performed using GraphPad Prism software. Hazard ratios were determined using growth as an event (designated as 1) and no growth was censored (designated as 0). In all cases wild type was considered as the reference for comparison. A positive hazard ratio suggested that the strain had higher evolvability than the wild type. The statistical significance of the model (based on p-values) was calculated using the Log-likelihood test. The hazard ratios and p-values from the model are reported in the text and figure legends.

### Calculation of epistasis and statistical significance using the error propagation method

Pairwise epistasis (ε) between two loci was calculated using a multiplicative model as described by Trindade and colleagues [77], as follows

$\epsilon =({\text{W}}_{\text{AB}}\;\times \;{\text{W}}_{\text{ab}})\;-\;({\text{W}}_{\text{aB}}\;\times \;{\text{W}}_{\text{Ab}})$

where, AB referred to wildtype, ab was the double mutant, and Ab/aB were the single mutants. The IC50 values of trimethoprim (representing fitness, W) were directly used to calculate pairwise epistasis (ε). If the double mutant (W_ab_) had a higher IC50 than the expected IC50 (W_aB_*W_Ab_), the epistatic interaction was positive, and if expected IC50 exceeded the observed IC50, the interaction was referred to as negative epistasis [78].

We used error propagation method to test if the epistasis was significantly different from zero. Error σε was given by:

$\epsilon =({\text{W}}_{{\text{ab}}^{\text{2}}}\times \sigma {\text{W}}_{{\text{AB}}^{\text{2}}}+\;{\text{W}}_{{\text{AB}}^{\text{2}}}\times \sigma {\text{W}}_{{\text{ab}}^{\text{2}}}\;+\;{\text{W}}_{{\text{aB}}^{\text{2}}}\times {\text{W}}_{{\text{Ab}}^{\text{2}}}\;+\;{\text{W}}_{{\text{Ab}}^{\text{2}}}\times \sigma {\text{W}}_{{\text{aB}}^{\text{2}}})$

where, σ represents the standard deviation of the measured IC50 values.

Epistasis (ε) was significantly different from zero, if the calculated value of ε was greater than the error σε [77,79]**.**

### Comparisons across experimental groups

Welch’s *t* test was used to compare different experimental groups unless otherwise mentioned. A *p*-value cut-off for statistical significance was set at 0.05 for all comparisons.

## Supporting information

S1 Fig PhoP-promoter activity in minimal media. Activity of the PhoP promoter measured using a GFP reporter in wild type (MG1655), *phoQ* knockout (ΔphoQ), *mgrB* knockout (ΔmgrB) and PhoQ knock-ins (V382G and A20P). Cultures were grown overnight in M9 media supplemented with indicated concentrations of MgSO_4_. GFP fluorescence (arbitrary units) was normalised to growth (optical density at 600 nm). Mean ± SD are plotted as bars and individual data points are overlaid. The data underlying this Figure are tabulated below the graph.
(TIFF)

S1 File Polymorphisms in the PhoQ-PhoP-DHFR-MgrB pathway identified from genome sequences of *E. coli* strains available in the NCTC database. (XLSX)

S2 File Raw numerical data used for plotting graphs in Figs 2–5. (XLSX)

S3 File Genomes sequencing of *E. coli* wild type or the DHFR-Trp47Arg knock-in strain evolved in trimethoprim (500 ng/mL). (XLSX)

S4 File RNA-sequencing of wild type or mutant *E. coli* strains. For all mutants, wild type *E. coli* was treated as reference.
(XLSX)

S5 File Raw numerical data from pairwise strain competition experiments used for plotting graphs in Fig 7. (XLSX)

S6 File List of oligonucleotides and PCR primers used in this study. (XLSX)

S1 Raw Image Uncropped immunoblot images for Fig 3C, 4B, 4H, 5I, and 5J. (TIFF)

## Abbreviations

AMR: antimicrobial resistance
CFU: colony-forming units
DHFR: Dihydrofolate Reductase
FDR: false discovery rate
TMP: trimethoprim.
