# Supplementary material for: Polymorphisms in a bacterial signalling pathway alter evolutionary routes of antibiotic resistance in Escherichia coli
Source: PLoS Biol. 2026 Aug 26;24(8):e3003977. doi: 10.1371/journal.pbio.3003977 (PMC13529223; doi:10.1371/journal.pbio.3003977)
Supplement: Supplementary file 1 [file pbio.3003977.s009.docx]

Reviewer #1:

Yelpure and colleagues report a series of experiments that explore the phenotypic consequences of single amino acid substitutions in the sensor kinase PhoQ and the diphydrofolate reductase as well as removal of MgrB, a negative regulator of the PhoQ protein, all from Escherichia coli. The manuscript is clearly written. It focuses on the impact that these mutations and deletion of the mgrB gene have in resistance to tripmethoprim and in fitness in media containing different concentrations of trimethoprim. The data are set up and interpreted in evolutionary terms. However, the physiological or clinical significance of the findings is unclear. Some of the statements made by the authors are not supported by the literature or the authors' own results. Certain experiments lack controls or are poorly designed. My specific comments are as follows:
Line 14: Evolvability of drug resistance can result from mutations in a variety of genes, not just those specifying a drug target or a detoxifying protein.

Response: We thank the reviewer for point this out. To be more correct, we have modified the statement to “Antibiotic resistance in bacteria frequently evolves due to mutations in drug-target or detoxifying genes”

Line 19: The authors' statement "When challenged with TMP, E. coli evolves to de-repress" goes against (neo)Darwinian evolution concept. In other words, there is no pre-determined goal in evolution.

Response: We have modified the statement to “When challenged with TMP, de-repression of the PhoQ-PhoP two-component signaling system in *E. coli* transcriptionally upregulates DHFR expression, leading to drug resistance.”

Line 32: The authors' statement "demonstrates that pre-existing polymorphisms alter both, evolvability and mutation landscapes during antibiotic adaptation." is obvious.

Response: We do not agree that this statement is obvious. Indeed, our own work shows that not all pre-existing polymorphisms in PhoQ lead to altered drug resistance (Fig. 1 and 2). In fact, most polymorphisms are expected to be near neutral and epistasis between them and adaptive mutations is, in our opinion, not necessary. Therefore, we have not modified our original statement.

Line 126: What is the phylogenetic relationship among the strains from which the investigated proteins originated?

Response: We have not investigated the phylogenetic relationship among the strains used in the analysis. However, the strains used for our analysis belonged to different serotypes and hence were likely to include phylogenetically diverse *E. coli*. We have included this information in the Supplementary File 1.

Line 130: The reason the authors provide for excluding MgrB from the analysis is not satisfactory given that this protein has been implicated in trimethoprim resistance.

Response: We have now included MgrB protein sequences from NCTC-strain collection for our analysis. The list of variations in MgrB, the distribution and predicted folding energy have been added to Supplementary File 1. However, we continue to feel that there are gaps in annotation and sequencing of small proteins like MgrB. Therefore we do not feel confident to follow up on variants detected.

Line 139: It is not surprising that the largest number of polymorphisms in PhoQ would map to the kinase domain as mutations in the regions in involved in interaction with PhoP may either kill the signal transduction pathway or result in non-physiological cross-talk with other two-component systems, which has been shown to be detrimental.

Response: It was our reasoning that since the kinase domain is the catalytic site, as the reviewer correctly points out, variation in this domain would have the highest probability of functional change. Given that PhoQ regulates a variety of cellular processes including pH and Magnesium homeostasis, it is expected that this domain would be under highest purifying selection. Thus, the observation that many sequence variants were present in this domain is, in our opinion, counter-intuitive and interesting.

Line 141: Provide references for the statement regarding the named sensors. Also, what about sensors no implicated in antibiotic resistance?

Response: We have updated references for sensors implicated in antibiotic resistance. Since our rationale was to study the effect of natural variation on antibiotic resistance, we did not look at polymorphisms in sensors that were not implicated in resistance. This is likely to be an interesting analysis indeed, however, we think that it may be beyond of the scope of the present study.

Line 145: Is there an experimental validation to support the use of the proxy?

Response: Though this has been tested in multiple systems, we are best acquainted with studies on DHFR. Work from the Shakhnovich group (PMID: **27001833**) and our own work (PMID: **29871875**) have shown this to be true.

Line 163: Indicate origin of replication of plasmid and plasmid copy number.

Response: The pCA24N plasmid has modified pMB1 replication origin and is present in 300-400 copies per cell (PMID: **21173244**). We have added these details to Table 2 in the Methods section.

Line 172 and throughout the manuscript: The relevance of the paper is undermined by growing bacteria in LB broth, which neither reflects an environment normally inhabited by pathogenic E. coli (i.e., those that would be targeted by trimethoprim) nor reflects the low Mg2+ or mildly acidic pH conditions normally activating the PhoP-PhoQ system.

Response: Luria-Bertani broth has a relatively low magnesium concentration of around 30-40 µM (PMID: **26443723**). The PhoPQ two-component system is known to be active when concentrations of Mg are in this range (PMID: **17909183** PMID: **34191587**). Thus, it is expected that PhoPQ would be active when strains are grown in LB alone. This is also demonstrated in our own work by a decrease in PhoP promoter activity in the PhoQ-knockout as seen in Figure 5. To confirm that activation by PhoQ mutants is also seen in minimal media at different Mg^2+^ concentrations, we have now performed PhoP promoter measurements in M9 supplemented with three different Mg^2+^ concentrations. The data are provided below.

In line with our data from LB, both V382G and A20P activate the pathway at 0.1 mM Magnesium, but at higher Mg concentration A20P continues to be active, while PhoQ-wild type and V382G show inhibition. The reviewer will note that in minimal media the extent of activation in the MgrB-knockout is the highest, which contradicts our observations in LB. However, because of the fitness cost of A20P we observed that in minimal media this mutant grew to very low densities, often not beyond an OD of 0.2. As a result the data for GFP/OD are not as reliable for this strain in minimal media as in LB. We have therefore not added these new data to the manuscript. They do make the important point though that our experiments in LB hold true, at least in terms of responses to Magnesium and activation of the PhoQ pathway by various mutants.

Since pH is a completely different environmental axis of investigation, we think that it is out of the scope of the present study. However, we agree that it would be interesting to see if responses to pH differ between PhoQ mutants.

Line 195: It is misguided to ascribe intention to bacteria (being poised).

Response: We have modified this usage to ‘facilitating the evolution of resistance’ throughout the manuscript.

Line 205: The results were entirely expected.

Response: We are unclear on what the exact objection here is and hence have not modified anything in this section.

Line 266: The authors explore conditions of questionable physiological significance. They should investigate established PhoP-dependent phenotypes such as survival of low Mg2+ or acidic pH.

Response: Multiple papers showing that PhoQP activation leads to trimethoprim resistance in *E. coli* have been published over the past 5-6 years by our group and others. We have also shown that many of the mutations we observe in MgrB are found in clinical strains of *E. coli* and *K. pneumoniae*. While we understand that historically Mg^2+^ and pH have been associated with PhoQP, trimethoprim resistance through regulation of DHFR in *E. coli* is backed up with peer-reviewed publications. Hence, we do not agree that our experiments have a questionable basis. The reviewer may look at the following papers for greater context: PMID: 34591012, PMID: 38032208, PMID: 40073078, PMID: 34394028.

Line 323 paragraph: The authors failed to examine/consider the effects of that the PhoQ mutations have on PhoQ's phosphatase activity towards phosphorylated PhoP.

Response: Hyperactivity of the PhoQ-Ala20Pro mutant could result from increased kinase activity or diminished phosphatase activity, or both. As the reviewer rightly points out, our study does not demonstrate which of these have been affected in the Ala20Pro mutant. However, the goal of this work was not to understand the biochemical basis of activation, but rather to understand the physiological and fitness effects of such mutations. We have therefore not added any experimental data directly testing this idea. We hope to do so in follow-up studies, where we are examining a larger number of activating mutations in PhoQ.

It is noteworthy however that the lower protein level of Ala20Pro-PhoQ in the MgrB-knockout is unlikely to be related to the Phosphatase/Kinase balance of PhoQ, since in these experiments PhoQ is under a heterologous promoter and hence free from the transcriptional feedback loop.

Line 348: Please provide a citation supporting the notion that a phoQ knock-out strain is deregulated (vs being inactive to promote the phosphorylated state of PhoP).

Response: As the reviewer rightly pointed out, there is expected to be no/low phosphorylated PhoP in a PhoQ-knockout. This will in turn prevent is from binding to target promoters as has been shown in multiple studies, for instance PMID: **15569664, PMID: 15569664.** Loss of PhoP recruitment to promoters leading to different level of transcription is what we meant by “deregulation”. We do not see any conceptual problem here and hence have made no modifications in this regard.

Line 348: Do the authors mean "deregulated" or "derepressed"?

Response: We meant deregulated in this context. Derepression would be incorrect since PhoP is a dual activator/repressor. However, this section has been substantially modified in response to comments by other reviewers. As a result this point may no longer be relevant.

Line 393: The significance of these results is not clear.

Response: In these experiments, we have addressed whether different PhoQP activity levels are selected by different antibiotic pressures. This line of investigation is significant as it reveals the relationship between drug pressure and most preferred activation state of PhoQ. The rationale is presented early in the paragraph.

Figure 1A: What is the evidence that the E. coli PhoQ protein senses low pH via its periplasmic domain?

Response: Samuel Miller's group has shown that the PhoQ sensor domain is responsive to changes in pH. Using NMR spectra of the periplasmic domain of *Salmonella* PhoQ, the group has charactersied conformational changes at different pH. A model suggesting that PhoQ activation at low pH is due to increased flexibility of the sensor domain's helices α4 and α5 has also been proposed. Here are the references: PMID: **26002083, PMID: 17466620**.

Figure 2G and others: What is the purpose of investigated the effect of Mg2+ concentrations above 10 mM given that the concentration of free Mg2+ is ~1 mM? Also the decrease in promoter activity between 0 and 10 mM is only 30% for the wild-type, much lower than what is reported in the literature. The mutant decreases only 10%. Therefore, the mutation does impact Mg2+ sensing, which is in contrast to what the authors state.

Response: Since all three strains, PhoQ Val382Gly, mgrB, and PhoQ Ala20Pro, were activating mutants, we wanted to look at their response to Mg. Rather than attempt to address what the activity would be under physiological conditions, it was our intention to use Mg as a tool to interrogate how mutations in PhoQ alter signal transduction in the protein. Since PhoQ Ala20Pro was not inhibited even at 5 mM, the Mg concentration was sequentially increased to assess its response at higher Mg levels and to ensure saturation of the sensor domain. There is precedent for this kind of approach and the use of >10 mM Mg concentrations (PMID: **17909183**).

------------------------------------------------------


Reviewer #2:

Review of "Polymorphisms in a bacterial signalling pathway alter evolvability of antibiotic resistance". In this paper the authors investigate the role of signalling pathways in the evolution of antibiotic resistance. Based on their results, they argue that naturally occurring polymorphisms alter the ability of E.coli to evolve antibiotic resistance. This is an interesting topic, and the authors present an extensive combination of genetic manipulation, experimental evolution and transcriptomics. The topic of this article (drivers of evolvability) is novel and timely, and the approaches that they have used are quite novel in that their work provides greater mechanistic insights into the causes of variation in evolvability than most studies in this area. The discussion linking the results of this study into the broader picture of the role of  regulatory pathways in bacterial evolution is interesting and thought provoking.  I think that this paper has the potential to make a
good contribution to PLoS Biology after the authors have addressed some points below. Most of these are points that will require small changes to the analysis or text and I think that these revisions are unlikely to alter the conclusions of this work. My one major concern comes from the evolution experiments reported in this paper - the methods are too brief and it is possible that the authors have pseudoreplicated their experiments and/or that cross-contamination may have occurred during the experiment. Furthermore, the manuscript lacks statistical analysis of evolvabilityand it is not clear to me that the results of these experiments support the conclusions of the paper.

1)      Introduction. In the introduction the authors state that previous studies from the MacLean lab have shown that pre-existing polymorphisms play an important role in the evolution of antibiotic resistance, but that the molecular mechanisms underpinning this are incorrect (line 79-91). The literature in this field is still quite small,  but it is broader than suggested by the authors, and many of these studies have incorporated important mechanistic insights into understanding the the causes of evolvability (ie doi: 10.1038/s41564-022-01282-x; doi: 10.1073/pnas.2308029120; doi: 10.1038/s41559-018-0547-x; doi: 10.1038/s41467-020-17735-y). The authors should place their interesting and novel study within the context of this literature in both the introduction and discussion. The recent paper by Rubin et al., for example, provides a very thorough and mechanistic study of how polymorphisms in pathogen populations shape the evolution of resistance.

Response: We thank the reviewers for suggesting research articles relevant to our study. We have incorporated them into the introduction section of the manuscript.

2)      Calculation of epistasis (Figure 3B). The data presented by the authors strongly suggests that epistasis occurs between mgrB and PhoQVal382Gly, but I am not convinced by their calculations. In order to test for epistasis they need to calculate an expected IC50 for the double mutants that propagates the error in the measurement in the single mutants and then test if the double mutant value is different from this additive expectation, taking into account error in the estimate of the IC50 in the double mutant. Methods for this can be found inhttps://[doi.org/10.1371/journal.pgen.1000578](http://doi.org/10.1371/journal.pgen.1000578)

Response: We thank the reviewer for pointing us to this method. We have now calculated epistatic interaction between PhoQ-Val382Gly and *mgrB* as described in Trindade *et al.* Further, we have also used the error-propagation method to determine the significance of the epistatic interaction.

A significant positive epistasis was observed between PhoQ-Val382Gly and the loss of mgrB. This has been reported in Figure 3 B and in the main text. We have also performed the same analysis to determine epistatic interactions between PhoQ-Ala20Pro and mgrB. Here we find the epistasis to be negative and these results have been added to Figure 5H and to the main text. These statistical analyses firm up our original claims.

3)      Experimental evolution (Figure 3 and 4)The methods used to describe the evolution experiment are not clear, and I have a few outstanding issues:
a)      Was each replicate population derived from a different clone or did all of the replicated of a mutant/wt come from a single culture. This is a critical issue to address, as if a single culture is used to setup the experiment the replicates are not truly independent of each other. For example, any polymorphisms present in the parental culture could have influenced evolution in multiple replicates.
b)      One danger of doing experimental evolution in microtiter plates is the potential for cross-contamination between replicates. What steps did the authors take to prevent cross-contamination, and what data can they present to suggest that cross-contamination was not important in their experiment.
c)      Genomic data can help to resolve points 1 and 2. For example, did the authors detect different mutations in different replicates, or did they detecte the same mutatiosn multiple times, which may be indicative of either cross-contamination or "founder effects" if a single culture was used to seed the different replicates. I do not understand Figure 4G, but it suggests that the same mutatiosn were detected in multiple cultures.
d)      Lack of statistical test. The authors have not tested for differences in evolvability/survivability between the WT and mutants. Tests for this based on Cox's proportional hazard model can be found in <https://www.nature.com/articles/s41559-018-0547-x> but other methods could also be used, such as tests comparing proportions of survivivng endpoint cultures. Given the relatively low sample sizes of cultures and the low number of surviving cultures, I would be surprised if many of the reported differences were statistically significant.

Response: These are all important concerns and we thank the reviewer for bringing them up. To provide greater clarity on the first 2 issues, we have modified the methods section as follows:

“For laboratory evolution of trimethoprim resistance 15 replicates of DHFR-Trp_47_Arg knock-in and *E. coli* wild type were grown in the wells of a 96-well plate in LB supplemented with 500 ng/mL of trimethoprim for 12 growth cycles. Replicates were derived from independent cultures that originated from three different colonies of each genotype. Further, evolution was performed in 3 sets, each consisting of 5 replicates, to prevent cross-contamination. Each growth cycle was for 24 hours after which 10 % of the culture was passaged into fresh media. At the end of 12 passages, glycerol stocks were prepared for surviving lineages and stored at -80 °C. ”

As the reviewer will appreciate from the above description, substantial care was taken to ensure that replicates were independent and cross-contamination was avoided. For greater clarity we have modified Figure 4 G and split the immunoblotting and genomics data. From the new Figure 4 G and the associated Supplementary File 3, the reviewer will appreciate that the four surviving lineages of the DHFR W47R knock-in strain, as well as the 15 wild type-derived populations, indeed had different mutational signatures validating that they were independently replicating populations.

To address the statistical testing of evolvability, we have used the Cox proportional hazard model as suggested by the reviewer. Using this test we found that there was indeed a statistically significant difference in the evolvability of PhoQ-V382G at MIC_wt_ (Cox proportional-hazards ratio at MIC_wt_ β1 = 3.27, likelihood ratio = 6.948, p < 0.0084) and DHFR-W47R knock-ins (Cox proportional-hazards ratio at MIC_wt_ β1 = 0.1504, likelihood ratio = 4.482, p < 0.0343). We did not analyse the data at higher concentrations since the number of surviving replicates was low precluding statistical testing.

Details of the test and the calculated hazard ratios have been added to the method section and to the main text, respectively.

4)      PhoQ-Ala20Pro mutation. The authors observed this mutation in cultures that were selected under trimethoprim and they conclude that this is a novel resistance conferring mutation (line 264-266). I think that this represents an over-interpretation of their results, as they did not evolve populations in the absence of TMP selection or test the effect of this mutation on TMP resistance. The observation that this substitution occurs in a clinical isolate is interesting, but not conclusive in any way as most mutations present in clinical isolates probably do not reprent beneificial mutations and in this case you effectively have a single observation.

Response: We thank the reviewer for this comment. They are indeed correct that based on a single clinical datapoint alone and in the absence of LB-alone evolution it is not prudent to conclude that the A20P mutation is a novel resistance conferring mutation. However, since we have subsequently gone on to show, using a knock-in, that the mutation is sufficient to confer drug resistance, including elucidation of the molecular basis, we think that we are justified in our conclusion. Nevertheless, we have softened our claim in the section pointed out by the reviewer.

5)      RNASeq analysis.
a)      The methods sugges that a single culture of each of strain was used to setup three cultures that were then used for RNAseq analysis. If this is the case (and the methods are unclear on this point) then it could be argued that the different cultures used are not true biological replicates. This point needs to be addressed.
b)       The authors identified differentially expressed genes as those with a least a 2 fold change in expression. This is not an acceptable methods, particularly given that error is expression measurements differs between genes. The authors need to use a method that identifies genes with statistically significant differences in expression using test that correct for multiple comparisons (line 339-344). The data presented seem to support the authors main conclusions (ie that the naturally occurring polymorphism leads to small changes in gene expression) but the differential gene expression analysis needs to be done correctly.

Response:

a)RNA sequencing for the strains was performed in three biological replicates originating from three independent cultures. We have added this clarification in the methods section.

b) We agree with the reviewer that statistical significance needs to be taken into account for gene expression changes. We have now reanalysed differentially expressed genes across strains using the adjusted p-value. The adjusted p-value corrects for multiple comparisons using the Benjamin-Hochberg method, and the cut-off is set to 0.05. Therefore, genes having log FC≥2 and p-adj≤0.05 are classified as upregulated, and log FC≥-2 and p-adj≤0.05 are referred to as downregulated in the updated analysis. This has been indicated in the methods section.

Indeed, we found that the PhoQ-Val382Gly knock-in had no significantly altered genes cementing our original claim that this polymorphism was mildly activating. However, after reanalysis, we found that many of “genome-wide” effects (originally Figure 6A) in other strains did not make the cut. Additionally, in an attempt to simplify this section of the manuscript as recommended by Reviewer 4, we have now focussed only on PhoP-regulated genes (primary and secondary targets) for this figure. These genes were indeed statistically significantly different in the *mgrB*-knockout and the PhoQ-Ala20Pro knock-in. As the reviewer will note, by and large our main conclusions are unaffected in the reanalyzed data.

6)      TMP co-culture experiments
a)      The authors report the results of their co-culture experiments in terms of the frequency of the competing strains. They report cases where all competing strains have a frequency of 0 - this is a result that has no logical interpretation, and it suggests that all of the bacteria in the culture have been killed. Based on their results, it looks like two different thigns in this experime are occurring: the relative abundance of the strains is changing as a result of competition and second the overall density of the culture is changing. One way to resolve this is to plot the density of the competing strains at each concentration of TMP, and this should capture both of the above effects. Alternatively, they could present data on total population density and fitness; for example as in <https://pmc.ncbi.nlm.nih.gov/articles/PMC5955900/>

Response: We thanks the reviewer for this observation. Indeed, both the relative abundance and the total density of the population do change with trimethoprim concentration. To avoid ambiguity, Figure 7 has been modified, and total cell density of the culture for each competition (i.e. total CFU/mL) is plotted as a bar graph on the right Y-axis. We believe that this representation is more meaningful.

7)      Line 419-422: "while at higher pressures benefit primarily determined the outcome." The use of benefit is unclear, do the authors mean MIC or resistance?

Response: We have changed the term ‘benefit’ to ‘resistance level’ for greater clarity

------------------------------------------------------------------------


Reviewer #3:

This manuscript by Matange and colleagues describes how the natural polymorphisms in the PhoQ-PhoP-DHFR pathway modulate both intrinsic TMP susceptibility and evolvability of resistance. One particular variant of PhoQ, Val382Gly (activating mutation), enhances evolvability through positive epistasis with mutations in mgrB. The Trp47Arg variant of DHFR reduces evolvability through negative epistasis with resistance mutations. This work systematically demonstrates that cost-benefit trade-offs determine optimal pathway activity during evolution. Specifically, the fitness cost from cross-activation of RpoS limits the evolution of high-activity PhoQ mutations. The optimal PhoQ activity is determined by the balance between benefit and cost, which varies with drug concentration. The data/results presented here strongly support the conclusions made by the authors. The work provides a mechanistic insight into how polymorphisms in a signaling pathway shape evolutionary trajectories
for drug resistance. Overall, this work is highly significant for evolutionary microbiology and antimicrobial resistance research.
I have a few comments, and the authors may also consider some of the suggestions, which I firmly believe would strengthen this paper.
Comments:
1.      In general, the authors provide a good literature context in the paper. It would be good to include Lenski group's work on historical contingency in evolution. Recent work on epistasis and evolutionary trajectories (from e.g., the Kryazhimskiy lab) could be referenced

Response: We have added both citations to the revised manuscript.

2.      Lines 325-326: figure citation is missing

Response: We have added the citation to the revised manuscript.

3.      Some of the bar graphs are missing statistical information (p-values, n values etc.)

Response: We have added this information to the revised manuscript.

4.      In terms of the mechanism, it is intriguing how MgrB may stabilize PhoQ-Ala20Pro.. In Fig. 5J, it appears that the Ala20Pro mutation destabilizes PhoQ by itself (as you can see stronger bands for both PhoQ WT and Ala20Trp mutant), and in the absence of MgrB, the mutant shows an even weaker band. On the contrary, in the absence of MgrB, both PhoQ WT and Ala20Trp mutant levels seem to be slightly higher than in the presence of MgrB. It has been proposed that PhoQ-Ala20Pro is directly/indirectly stabilized by MgrB. A structural model of PhoQ-Ala20Pro with MgrB could help explain the dependence on MgrB. A few technical details are missing in the methods. How are the cells grown for the immunoblotting experiments? are the cells used here in the exponential or the stationary phase? Since Ala20 is in the TM region is it possible that the PhoQ variant is not localized as efficiently to the membranes? The authors should analyze membrane and cytosolic fractions separately as
opposed to total cell lysates for each PhoQ variant.

Response: We have added details on growth conditions and duration to the methods section.

In line with the reviewer’s suggestion, we have now assessed whether the Ala20Pro and Ala20Trp mutants mislocalise to the cytosol. For this, we prepared sub-cellular fractions as follows:

Overnight cultures were diluted 1:100 into 6 mL LB and grown for 3 hours at 37°C. Cells were pelleted and resuspended in 500 µL of lysis buffer. Cell lysis was performed using sonication. The lysate was centrifuged at 1000 g to separate out the unlysed cells. The supernatant was then subjected to ultracentrifugation at 1,00,000 g to obtain cytosolic and membrane fractions. The membrane pellet was resuspended in 500 µL of lysis buffer and homogenized by brief sonication. Protein concentration was estimated using the Bradford method. A total of 10 µg of the whole-cell lysate protein and volumetrically equivalent cytosol and membrane fractions were used for immunoblotting using an anti-His primary antibody to detect heterologously expressed PhoQ.

Apart from a decrease in overall expression we don’t see a mis-localisation of either of the mutants. Since the manuscript is already data dense we have not added these data to the revised version. However, a representative blot is provided below for the reviewer’s benefit.

5.      Number of replicates for the transcriptome analysis by RNA-Seq not mentioned in the main text.

Response: We have modified the methods section for RNA seq to include the missing details. There were 3 independent biological replicates.

6.      It might be worth testing whether an rpoS deletion in PhoQ-Val382Gly cells changes the outcome of the competition assay, in terms of cost compensation.

Response: We did consider performing this experiment. However, since PhoQ-Val382Gly did not have a fitness cost to begin with (Figure 6), we hypothesize that deletion of *rpoS* is less likely to affect the outcome of the competition, irrespective of the competing strain. Therefore we have not performed this experiment.

------------------------------------------------------------------------


Reviewer #4:

In this manuscript "Polymorphisms in a bacterial signalling pathway alter evolvability of antibiotic resistance", Yelpure and colleagues ask how standing genetic variation in a signally pathway can alter both that pathway's workings and potential to evolve in response to selective pressure. The authors identified several polymorphisms found in E. coli in the PhoQ-P-DHFR pathway, and characterised the potential of these mutations to alter DHFR transcription and resistance to trimethoprim. The authors then focus on a subset of mutations and measure how these mutations affect evolvability, in both the types of mutations that confer resistance and their level of conferred resistance. One of these polymorphisms allowed a rarely reported PhoQ mutant with high resistance yet high-costs. The authors detail the general transcriptomic effects of this phoQ mutation that explain the rare evolution of this particular genotype. Provided, is a nice detailed example of how subtle
polymorphisms can have marked regulatory changes which ultimately affect the potential evolvability of a strain, even with a selective agent like Trimethoprim, which should confer a selective pressure with a limited number for mutational 'responses'.

The paper is clearly written, the experimental work convincing and detailed, and the conclusions that genetic variation alters evolvability is supported by the data. The concept that mutations may interact and alter evolutionary trajectories is as old as the concept of epistasis, but the authors do make a nice contribution by detailing the mechanistic sources of epistasis (at least in this signalling system). My comments (below) are mostly points that require clarification in the text. One fundamental issue - which the authors must address - is the extent to which these are really "polymorphisms" in the phoQ pathway. The authors should confirm in the manuscript that in the isolated polymorphisms, that there are no secondary mutations in the PhoQ-P-DHFR pathway that would alter the activity of the pathway. Otherwise, claims that these polymorphisms alter evolutionary trajectories would be out of context, given these polymorphisms may be linked to secondary mutations which
alter their phenotypes. I would also comment that while I like the level of detail presented, the authors could really aim to remove sections of the text to some kind of supplement (for instance, I think a lot of the RNA seq data is simply superfluous to the central story). The same goes for many panels in the figures (see comments of figures below). This would allow greater attention to be made to the more central findings and claims and better annotations of the figures (which could be enlarged and made easier to understand). Regardless of the presentation, I commend the authors on their deep dive into their system and I appreciate the many ways in which they analysed mutational effects.


Specific comments/questions are below.

Line 128. It would be important to state here (early on) or otherwise clearly show that these polymorphisms are not found in combination with other polymorphisms in the Pho pathway. As these mutations are individually knocked it, that could divorce them from other mutations which in combination alter their phenotype. If these mutations are found in combination, best practice would be to knock in multiple mutations to assess their effects on evolvability.

Response: This is indeed a very important point, and we thank the reviewer for taking note of it. We have now added a strain-wise identification of polymorphisms in the PhoQ-PhoP-DHFR pathway in Supplementary File 1. In response to the reviewer’s comment we make the following observations:

1. While many PhoQ variants were stand-alone some did indeed occur with other variants in the pathway. The most common was the I44L polymorphism in PhoP. However, roughly ~50% of the strains analysed by us had this polymorphism. Given that it is a conservative substitution (predicted ΔΔG = -0.99 kcal/mol) we surmise that this is most likely a neutral variant.
2. Some PhoQ polymorphisms co-occurred with each other. R328H, E464D and A482T often appeared in the same strain. Yet, there were instances of these polymorphisms in isolation too. Thus, while their functional effects may impact each other, the rationale to bring them into the lab strain as a stand-alone mutation still holds.
3. The two main polymorphisms that we pursued in the study, V382G in PhoQ and W47R in DHFR, did occur as stand alone in addition to co-occurring with variants in other variants proteins of the pathway. It would indeed be interesting to look at the effects of combinations of co-occuring polymorphisms on the evolvability. However, from the point of view of this study, the rationale to bring them into the lab strain as a stand-alone mutation still holds.

Line 168: Define jargon like "IC50" on first usage.

Response: Done.

Line 170 "Further, the promoter activity of PphoP and PfolA, both responsive to active PhoP," provide citation for this statement.

Response: We have added citations to the statement in the revised manuscript.

Line 216 "Strikingly, combining Trp47Arg with at least 4 different resistance-conferring mutations in DHFR substantially diminished TMP resistance". Please articulate in the text why this was done.

Response: The motivation here was to understand whether pre-existing polymorphs can modify the phenotype of resistant mutations in DHFR due to intragenic epistasis. We have added the rationale for studying trimethoprim resistance of the double mutants in the revised manuscript.

Line 308: Please provide a broad conclusion to this paragraph - it's not clear to me what to take away from these Magnesium experiments.

Response: Magnesium is an inhibitor of PhoQ that binds extracellularly and drives the kinase domains into an inactive conformation. We wanted to check if A20P locked the protein in an active conformation, and whether the mutant protein was capable of sensing Mg. Our results showed that the A20P mutant protein did respond to Mg level, but had a higher activity than wild type even at saturating Mg. Thus, it could be argued that the mutation locks the kinase in an active conformation, but remains able to sense magnesium levels.

We have added the following line to the revised manuscript for greater clarity:

“These data indicated that the Ala20Pro mutation altered the basal activity of PhoQ, possibly by inducing a conformational change that locked the kinase domains in an active conformation.”

Lines 323 to 335: I was confused by this paragraph - I initially thought the PhoQ-Ala20Pro was influencing PhoQ transcription/translation, but later in the paragraph I realised the mutation affected protein stability. Perhaps it's better to refer to "protein level" in line 325 as opposed to expression-level, as expression level tends to invoke the level of creation of protein.

Response: We agree with the reviewer on this point, and we have explicitly mentioned ‘protein level’ in the revised manuscript.

Lines 337-382: I think there is simply too much information in this section. The point to get to is that PhoQ signalling induces a regulon like rpoS, and that might be costly (and you follow this up later). I know this RNA work is a lot of work, but best to have the results of Fig 6A-C in supplements and get to the main point (Fig D-F).

Response: We have substantially simplified the figure by restricting the analysis to PhoP-regulated genes.

Line 339: There is little explanation for why this RNA-work is done. Because of this, I just can't anticipate how this experimental work will fit into the narrative, which has been reasonably straight forward until now. Please introduce this section with a clear motivation. Furthermore, it's confusing to present both the polymorphic mutations and the mutations that evolved in the background of these polymorphisms at the same time. Best is to split these types of mutations up (and their RNA analyses) and avoid confusion.

Response: We have substantially simplified this section, and have also added a rationale for the RNA seq analysis.

This part of the study was motivated by our earlier findings that altering PhoQP activity by deleting MgrB alters a larger network of gene regulatory proteins that are downstream of PhoQ. We wanted to understand how this pervasive impact scaled with the level of activation of PhoQP by different mutations. We hope that this point comes across in the revised manuscript.

Line 353: Reference needed for statement "In E. coli, 55 protein coding genes are known to be direct targets of PhoP."

Response: Citation added to the revised manuscript.

Line 355: "However, de-repression or hyperactivation of PhoQ." Not specific - state which mutations this refers to.

Response: We have added the details in the main text for greater clarity.

Line 360: This paragraph misses a concluding statement - I'm not sure what to learn from it.

Response: We concluded from this section that even though mutations may activate PhoQP to different extents, targets of this pathway were not all similarly affected. We have added this conclusion to the revised manuscript.

Line 376: This line implies that rpoS was deleted in combination with PhoQ-ala20Pro, but this genotype is not in the Fig 6F. I assume then that one of the competitors is delta rpoS, but this is really not clear from the legend. Please carefully label the axis and provide more detail in the legend - It's not clear what the competitions are in Fig 6F.

Response: We apologies for this error, and thank the reviewer for pointing it out. We have corrected the labels in Fig 6D (old figure 6F).

Line 395: Can the authors please state why they are using these frequency measures when they could be using a simple fitness/selection coefficient measure?

Response: In competition experiments, there were events where one or both competing strains went extinct. This extinction was antibiotic concentration-dependent. If one of the competitors was not detected, it would make the fitness of the other strain infinite. We have therefore not used fitness for these analysis, rather maintained the data as frequency. For greater clarity, we have also provided the total CFU/mL in the revised manuscript.

Line 439: "leftward shift": Please fully explain what this means (I know what it means, but the reference to the figure needs to be made clear).

Response: We have modified the sentence to “…demonstrated a substantial reduction in the lowest selective concentration of antibiotic.”

Line 505-6 and following lines : "Cross-activated". Why use the term cross-activated as opposed to simply activated? The term cross implied this is some kind of mistaken activation, which - given the cost of rpoS misregulation - is almost certainly not the case. Rather, the PhoQ signals are one of the many that influence regulation of rpoS.

Response: We agree that cross-activation seems to suggest that RpoS was misregulated and thank the reviewer for pointing this out. We meant that downstream effectors which are themselves transcription factors were activated by PhoP. We have modified the text accordingly.

Line 527- "breaks this barrier". This analogy is a little vague - rephrase and clarify what is meant.
Response: We have modified the sentence to “Ultimately the strength of selection for higher activity would determine whether a signalling pathway overcomes the cost of cross-activation during its evolution.”

Lines 529-555 - this is rather complicated paragraph and I'm not sure what to take away from it. Perhaps that the effects of Pho-pathway activation are so numerous, the source of "cost" of this activation is difficult to pinpoint. I would encourage the authors to really cut this section down and avoid unnecessary speculation.

Response: The purpose of this paragraph was to suggest a possible molecular explanation for why activating the PhoQP pathway to different extents has different outcomes for individual targets within the PhoP-regulon. While we agree that there is speculation involved we believe that putting these ideas down in black and white may allow others to examine their own systems in this light and ultimately lead to some insight. We have therefore not modified the section, but if the reviewer and editors feel that this section continues to be superfluous we can shorten/remove it from the discussion.


GENERAL FIGURE COMMENTS

Figures 5B, 6B, 6F. There is several applications of the students t-test. Can the authors please state what assessment was done of the assumptions of the t-test (esp. homogeneity of variance and normality), because some of the raw data do not look suitable for a t-test (unless the test was applied on a transformation of the data, in which case that data should be displayed in the plots).

Response:

Response: We thanks the reviewer for pointing this out. We have now analysed the data using Welch’s t-test to account for different variances. We have not explicitly tested for normality, but we do not anticipate any of the data to be not normally distributed.

Fig1-7: Generally, there are many plots in all the figures that could use more detail so that they are immediately understood. If you can simply add a word to a y-axis that lets the reader understand what the plot and the values measured, then this is preferable. For instance, Fig 2D is labelled "Fold over WT". There's no clue what was actually measured until you read the legend. I would argue "GFP expression rel. to WT" (or similar) would give a far better indication that these promoter studies use a GFP reporter to measure expression relative to a WT strain. This also applied to 4B (what's R/W?), 4D (Y-axis could be "relative fitness of DHFR-Trp47Arg"), 5C and 6F (both needing y-axis labelling).

Response: We have re-labelled the suggested figures for greater clarity.

Fig 1-7: I appreciate the work that's gone into these figures, but I'm encourage the authors to move some of these panels to supplements. Many of them are not crucial for the main points being made and distract from the message. I'm specifically referring to 1B (bottom green panel), 2E, 3C, 4B, 4G, 5D, 6A, 6B, 6C. Though this is more a point of style and not a crucial requirement, but I think it would really help the readers to engage with this paper.

We could move 1B heat map for other SKs, 4B, 5D, 6A (only deregulated genes and not the volcano plots)

Response: We understand the concern here, and struggled with it ourself. However, after much deliberation we have decided to retain some of the suggested panels. In part this is motivated by the worrying trend where bulk of data are in supplementary sections, which are, by and large, ignored or have a lower standard for rigour.

We have, however, simplified Figure 6 substantially as indicated earlier and hope that this will make understanding the manuscript easier.

Figure 8A: I appreciate the attempt to make a diagram on some of the more abstract concepts, but his diagram doesn't clarify things for me. What's the distinction of "novel mutations and adaptive mutations? Why is the cell with novel mutation the same colour as a mutation with lower evolvability. Best remove 8A or drastically alter it.

Response: In retrospect we understand how this figure may be very difficult to follow. In part, this was because of the complexity of the claims that we were trying to squeeze into a single model. We have simplified the figure to include only the major claims of the study. We hope that the revised figure has greater clarity.

Figure 8B: I don't understand the "plot" at the bottom. What is the y-axis supposed to mean? Why are such large error bars presented? This needs revision, though I would like to see the molecular map kept in the top half of 8A- but it needs to be made larger and more clearly annotated.

Response: Same as above.

__________________________________________________
