# Supplementary material for: Polymorphisms in a bacterial signalling pathway alter evolutionary routes of antibiotic resistance in Escherichia coli
Source: PLoS Biol. 2026 Aug 26;24(8):e3003977. doi: 10.1371/journal.pbio.3003977 (PMC13529223; doi:10.1371/journal.pbio.3003977)
Supplement: Supplementary file 3 [file pbio.3003977.s011.docx]

Response to reviewers’ and editors’ comments:

As you will see from the reports, the reviewers generally consider the revised manuscript to be substantially improved. However, the Academic Editor has identified several remaining issues that require careful attention before the manuscript can be considered further. In particular, the description of how conducting the evolution experiments in three sets prevented cross-contamination should be clarified, including how cross-contamination among the five replicate populations within each set was avoided.

Response: We have added these details to the methods section as well as the results section of the revised manuscript. We clarify why we chose to perform these evolution experiments as 3 sets of 5 replicates each, as well as how genome sequence *post facto* showed that the 15 replicates were indeed independent, despite convergent mutations in *mgrB* and *folA*.

Please also check the reporting and interpretation of the Cox proportional-hazards analyses, including whether values currently presented as thresholds, such as “p < 0.0084,” should instead be reported as exact P-values.

Response: We have revised the reporting of Cox proportional-hazards analyses to include hazard ratio β1, 95% CI, likelihood ratio as well as precise p-values. The p-values provided in the earlier draft were already the precise values and the “<” sign was a typographical error.

The distinction between altered “evolvability” and altered “mutation landscapes” should be explained more precisely, and vague or potentially overlapping terminology should be revised throughout.

Response: We appreciate the concern with regards to these terms. We have now revised the manuscript, especially in the discussion section, to avoid ambiguity.

In addition, the new PhoP promoter assay results obtained in M9
should be presented, at least in the supplementary material, and the apparent sensitivity of the mgrB knockout phenotype to assay conditions in LB versus M9 should be discussed; the lower optical density alone does not appear to justify describing these reproducible data as less reliable.

Response: We have added these data to Supplementary Figure 1.

Finally, please ensure consistent use of terminology when describing epistasis, as suggested by Reviewer #2. Addressing these points thoroughly and carefully is necessary for further consideration of the manuscript for publication in PLOS Biology.

Response: We have modified the terminology to make it more consistent as suggested by the reviewer. We use “positive and negative epistasis” throughout the revised manuscript.

----------------------------------------------------------------
REVIEWERS' COMMENTS:
----------------------------------------------------------------

Reviewer #2

The authors have addressed my previous concerns. One suggestion is that the authors refer to epistasis as positive/negative and antagonistic/synergistic. For simplicity, I suggest sticking with one set of terms throughout the manuscript.

Response: We thank the reviewer for their careful reading of the earlier draft.

We have now revised the manuscript to consistently use positive/negative epistasis, which is the least ambiguous terminology.

— — —

Reviewer #3

The revision addresses most of my concerns in the initial manuscript. Overall, this paper highlights how naturally occurring sequence variations in PhoQ change the ability of E.coli cells to evolve antibiotic resistance.

Response: We thank the reviewer for their careful consideration of our earlier draft.

— — —

Reviewer #4 (Andrew Farr)

This follows up on a previous review of submission "Polymorphisms in a bacterial signalling pathway alter evolvability of antibiotic resistance". I was largely happy with the first manuscript, except for one requirement that that the authors investigate the genotypic context (and potential epistatic interactions) of the main mutations that were analysed in this work. I also thought the manuscript could be refined further: some of the results were missing motivation or summary statements and many data plots were often insufficiently labelled.

Response: We thank the reviewer for his careful consideration of our earlier draft.

It was great to see the extra effort in resolving how many mutations are present in the environmental isolates. There is still a possibility the I44L mutation in PhoP could be altering the phenotypes resulting from mutations in either PhoQ and DHFR. However, I also appreciate that this is a model of evolutionary processes, not a replication of how the genetic pathway might be evolving in the 'wild' - which would of course require different experimental conditions. Importantly, the focal mutations can be found in the absence of other intra-protein mutations (i.e. they are not in combination with other mutation/s to that protein). The authors make a reasonable suggestion that these focal mutations affect protein stability and activity, both in the isolates and in the experimental reconstructed mutants. In any case - given the emphasis on the impact of polymorphisms on altering mutational trajectories - it's been an important addition to provide some perspective on how much (or
rather, how little) genetic diversity there is across the E coli isolates.

Response: Indeed, it would be very interesting to see what the evolvability of the “wild strain” is and one possibility would be to back mutate the I44L position in the original strain. However, as the reviewer notes, this is beyond the scope of the present study.

Otherwise, I'm happy with the many clarifications/elaborations within the text (which mainly help to contextualise the results), the additional labelling to plots, additional data added to plots, the application of more suitable statistics and the removal of some panels (especially in Figures 6 and 8). I'm happy to recommend publication of this manuscript without further modification.

Response: Thank you!
