# Supplementary material for: Polymorphisms in a bacterial signalling pathway alter evolutionary routes of antibiotic resistance in Escherichia coli
Source: PLoS Biol. 2026 Aug 26;24(8):e3003977. doi: 10.1371/journal.pbio.3003977 (PMC13529223; doi:10.1371/journal.pbio.3003977)
Supplement: Peer review history

# Author comment: Author response to Decision Letter 0

**DOI:** 10.1371/journal.pbio.3003977.r001

2 May 2026

Attachment Submitted filename: Response to reviewers_NM.docx

# Editor report: Decision Letter 0

**DOI:** 10.1371/journal.pbio.3003977.r002

**Authors:** Melissa Vazquez Hernandez

12 May 2026

Dear Nishad,

Thank you for submitting your revised manuscript entitled "Polymorphisms in a bacterial signaling pathway alter evolvability of antibiotic resistance" for consideration as a Research Article by PLOS Biology.

Your manuscript has now been evaluated by the PLOS Biology editorial staff as well as by the previous academic editor with relevant expertise and I am writing to let you know that we would like to send your submission back to the original reviewers.

However, before we can send your manuscript to reviewers, we need you to complete your submission by providing the metadata that is required for full assessment. To this end, please login to Editorial Manager where you will find the paper in the 'Submissions Needing Revisions' folder on your homepage. Please click 'Revise Submission' from the Action Links and complete all additional questions in the submission questionnaire.

Once your full submission is complete, your paper will undergo a series of checks in preparation for peer review. After your manuscript has passed the checks it will be sent out for review. To provide the metadata for your submission, please Login to Editorial Manager (https://www.editorialmanager.com/pbiology) within two working days, i.e. by May 14 2026 11:59PM.

If your manuscript has been previously peer-reviewed at another journal, PLOS Biology is willing to work with those reviews in order to avoid re-starting the process. Submission of the previous reviews is entirely optional and our ability to use them effectively will depend on the willingness of the previous journal to confirm the content of the reports and share the reviewer identities. Please note that we reserve the right to invite additional reviewers if we consider that additional/independent reviewers are needed, although we aim to avoid this as far as possible. In our experience, working with previous reviews does save time.

If you would like us to consider previous reviewer reports, please edit your cover letter to let us know and include the name of the journal where the work was previously considered and the manuscript ID it was given. In addition, please upload a response to the reviews as a 'Prior Peer Review' file type, which should include the reports in full and a point-by-point reply detailing how you have or plan to address the reviewers' concerns.

During the process of completing your manuscript submission, you will be invited to opt-in to posting your pre-review manuscript as a bioRxiv preprint. Visit http://journals.plos.org/plosbiology/s/preprints for full details. If you consent to posting your current manuscript as a preprint, please upload a single Preprint PDF.

Feel free to email us at plosbiology@plos.org if you have any queries relating to your submission.

Kind regards,

Melissa

Melissa Vazquez Hernandez, Ph.D.

Associate Editor

PLOS Biology

mvazquezhernandez@plos.org

# Author comment: Author response to Decision Letter 1

**DOI:** 10.1371/journal.pbio.3003977.r003

12 May 2026

Attachment Submitted filename: Response_to_reviewers_NM_auresp_1.docx

# Peer review: Decision Letter 1

**DOI:** 10.1371/journal.pbio.3003977.r004

**Authors:** Melissa Vazquez Hernandez

15 Jul 2026

Dear Nishad,

Thank you for your patience while we considered your revised manuscript "Polymorphisms in a bacterial signaling pathway alter evolvability of antibiotic resistance" for consideration as a Research Article at PLOS Biology. Your revised study has now been evaluated by the PLOS Biology editors, the Academic Editor and three out of the four original reviewers.

As you will see from the reports, the reviewers generally consider the revised manuscript to be substantially improved. However, the Academic Editor has identified several remaining issues that require careful attention before the manuscript can be considered further. In particular, the description of how conducting the evolution experiments in three sets prevented cross-contamination should be clarified, including how cross-contamination among the five replicate populations within each set was avoided. Please also check the reporting and interpretation of the Cox proportional-hazards analyses, including whether values currently presented as thresholds, such as “p < 0.0084,” should instead be reported as exact P-values. The distinction between altered “evolvability” and altered “mutation landscapes” should be explained more precisely, and vague or potentially overlapping terminology should be revised throughout. In addition, the new PhoP promoter assay results obtained in M9 should be presented, at least in the supplementary material, and the apparent sensitivity of the mgrB knockout phenotype to assay conditions in LB versus M9 should be discussed; the lower optical density alone does not appear to justify describing these reproducible data as less reliable. Finally, please ensure consistent use of terminology when describing epistasis, as suggested by Reviewer #2. Addressing these points thoroughly and carefully is necessary for further consideration of the manuscript for publication in PLOS Biology.

In addition to these revisions, you may need to complete some formatting changes, which you will receive in a follow up email. A member of our team will be in touch with a set of requests shortly. If you do not receive a separate email within a few days, please assume that checks have been completed, and no additional changes are required.

We expect to receive your revised manuscript within 1 month. Please email us (plosbiology@plos.org) if you have any questions or concerns, or would like to request an extension.

At this stage, your manuscript remains formally under active consideration at our journal; please notify us by email if you do not intend to submit a revision so that we withdraw the manuscript.

**IMPORTANT - SUBMITTING YOUR REVISION**

Your revisions should address the specific points made by each reviewer. Please submit the following files along with your revised manuscript:

1. A 'Response to Reviewers' file - this should detail your responses to the editorial requests, present a point-by-point response to all of the reviewers' comments, and indicate the changes made to the manuscript.

*NOTE: In your point-by-point response to the reviewers, please provide the full context of each review. Do not selectively quote paragraphs or sentences to reply to. The entire set of reviewer comments should be present in full and each specific point should be responded to individually.

You should also cite any additional relevant literature that has been published since the original submission and mention any additional citations in your response.

2. In addition to a clean copy of the manuscript, please also upload a 'track-changes' version of your manuscript that specifies the edits made. This should be uploaded as a "Revised Article with Changes Highlighted " file type.

*Resubmission Checklist*

When you are ready to resubmit your revised manuscript, please refer to this resubmission checklist: https://plos.io/Biology_Checklist

To submit a revised version of your manuscript, please go to https://www.editorialmanager.com/pbiology/ and log in as an Author. Click the link labelled 'Submissions Needing Revision' where you will find your submission record.

Please make sure to read the following important policies and guidelines while preparing your revision:

*Published Peer Review*

Please note while forming your response, if your article is accepted, you may have the opportunity to make the peer review history publicly available. The record will include editor decision letters (with reviews) and your responses to reviewer comments. If eligible, we will contact you to opt in or out. Please see here for more details:

https://blogs.plos.org/plos/2019/05/plos-journals-now-open-for-published-peer-review/

*PLOS Data Policy*

Please note that as a condition of publication PLOS' data policy (http://journals.plos.org/plosbiology/s/data-availability) requires that you make available all data used to draw the conclusions arrived at in your manuscript. If you have not already done so, you must include any data used in your manuscript either in appropriate repositories, within the body of the manuscript, or as supporting information (N.B. this includes any numerical values that were used to generate graphs, histograms etc.). For an example see here: http://www.plosbiology.org/article/info%3Adoi%2F10.1371%2Fjournal.pbio.1001908#s5

*Blot and Gel Data Policy*

We require the original, uncropped and minimally adjusted images supporting all blot and gel results reported in an article's figures or Supporting Information files. We will require these files before a manuscript can be accepted so please prepare them now, if you have not already uploaded them. Please carefully read our guidelines for how to prepare and upload this data: https://journals.plos.org/plosbiology/s/figures#loc-blot-and-gel-reporting-requirements

*Protocols deposition*

To enhance the reproducibility of your results, we recommend that if applicable you deposit your laboratory protocols in protocols.io, where a protocol can be assigned its own identifier (DOI) such that it can be cited independently in the future. Additionally, PLOS ONE offers an option for publishing peer-reviewed Lab Protocol articles, which describe protocols hosted on protocols.io. Read more information on sharing protocols at https://plos.org/protocols?utm_medium=editorial-email&utm_source=authorletters&utm_campaign=protocols

Thank you again for your submission to our journal. We hope that our editorial process has been constructive thus far, and we welcome your feedback at any time. Please don't hesitate to contact us if you have any questions or comments.

Sincerely,

Melissa

Melissa Vazquez Hernandez, Ph.D.

Senior Editor

PLOS Biology

mvazquezhernandez@plos.org

----------------------------------------------------------------

REVIEWERS' COMMENTS:

----------------------------------------------------------------

Reviewer #2

The authors have addressed my previous concerns. One suggestion is that the authors refer to epistasis as positive/negative and antagonistic/synergistic. For simplicity, I suggest sticking with one set of terms throughout the manuscript.

— — —

Reviewer #3

The revision addresses most of my concerns in the initial manuscript. Overall, this paper highlights how naturally occurring sequence variations in PhoQ change the ability of E.coli cells to evolve antibiotic resistance.

— — —

Reviewer #4 (Andrew Farr)

This follows up on a previous review of submission "Polymorphisms in a bacterial signalling pathway alter evolvability of antibiotic resistance". I was largely happy with the first manuscript, except for one requirement that that the authors investigate the genotypic context (and potential epistatic interactions) of the main mutations that were analysed in this work. I also thought the manuscript could be refined further: some of the results were missing motivation or summary statements and many data plots were often insufficiently labelled.

It was great to see the extra effort in resolving how many mutations are present in the environmental isolates. There is still a possibility the I44L mutation in PhoP could be altering the phenotypes resulting from mutations in either PhoQ and DHFR. However, I also appreciate that this is a model of evolutionary processes, not a replication of how the genetic pathway might be evolving in the 'wild' - which would of course require different experimental conditions. Importantly, the focal mutations can be found in the absence of other intra-protein mutations (i.e. they are not in combination with other mutation/s to that protein). The authors make a reasonable suggestion that these focal mutations affect protein stability and activity, both in the isolates and in the experimental reconstructed mutants. In any case - given the emphasis on the impact of polymorphisms on altering mutational trajectories - it's been an important addition to provide some perspective on how much (or rather, how little) genetic diversity there is across the E coli isolates.

Otherwise, I'm happy with the many clarifications/elaborations within the text (which mainly help to contextualise the results), the additional labelling to plots, additional data added to plots, the application of more suitable statistics and the removal of some panels (especially in Figures 6 and 8). I'm happy to recommend publication of this manuscript without further modification.

# Author comment: Author response to Decision Letter 2

**DOI:** 10.1371/journal.pbio.3003977.r005

29 Jul 2026

Attachment Submitted filename: Response to Reviewers.docx

# Editor report: Decision Letter 2

**DOI:** 10.1371/journal.pbio.3003977.r006

**Authors:** Melissa Vazquez Hernandez

5 Aug 2026

Dear Nishad,

Thank you for your patience while we considered your revised manuscript "Polymorphisms in a bacterial signaling pathway alter evolvability of antibiotic resistance" for publication as a Research Article at PLOS Biology. This revised version of your manuscript has been evaluated by the PLOS Biology editors and the Academic Editor.

Based on our Academic Editor's assessment of your revision, we are likely to accept this manuscript for publication, provided you satisfactorily address the following data and other policy-related requests.

1) We routinely suggest changes to titles to ensure maximum accessibility for a broad, non-specialist readership, and to ensure they reflect the contents of the paper. In this case, we would suggest a minor edit to the title, as follows. Please ensure you change both the manuscript file and the online submission system, as they need to match for final acceptance:

"Polymorphisms in the PhoQP signaling pathway alter evolutionary routes of antibiotic resistance in E. coli"

2) We noticed that in the abstract you still use "evolvability" and "mutation landscape". Just wanted to let you know in case you want to adjust it now.

3) You may be aware of the PLOS Data Policy, which requires that all data be made available without restriction: http://journals.plos.org/plosbiology/s/data-availability. For more information, please also see this editorial: http://dx.doi.org/10.1371/journal.pbio.1001797

Please supply the numerical values either in the a supplementary file or as a permanent DOI’d deposition for the following figures:

Figure 1BC, 2ABCDFG, 3ABE, 4ACDE, 5A-H, 6ACD, 7B-I, S1

NOTE: the numerical data provided should include all replicates AND the way in which the plotted mean and errors were derived (it should not present only the mean/average values).

4) Please cite the location of the data clearly in all relevant main and supplementary Figure legends, e.g. “The data underlying this Figure can be found in S1 Data” or “The data underlying this Figure can be found in https://doi.org/10.5281/zenodo.XXXXX”

5) We require the original, uncropped and minimally adjusted images supporting all blot and gel results reported in the Figures 3C, 4BH, 5IJ

-- We will require these files before a manuscript can be accepted so please prepare and upload them now. Please carefully read our guidelines for how to prepare and upload this data: https://journals.plos.org/plosbiology/s/figures#loc-blot-and-gel-reporting-requirements

6) Please ensure that your Data Statement in the submission system accurately describes where your data can be found and is in final format, as it will be published as written there

7) Per journal policy, if you have generated any custom code during the course of this investigation, please make it available without restrictions. Please ensure that the code is sufficiently well documented and reusable, and that your Data Statement in the Editorial Manager submission system accurately describes where your code can be found. More information on our Code Policy, what and how to share can be found here: https://journals.plos.org/plosbiology/s/code-availability

Please note that we cannot accept sole deposition of code in GitHub, as this could be changed after publication. However, you can archive this version of your publicly available GitHub code to Zenodo. Once you do this, it will generate a DOI number, which you will need to provide in the Data Accessibility Statement (you are welcome to also provide the GitHub access information). See the process for doing this here: https://docs.github.com/en/repositories/archiving-a-github-repository/referencing-and-citing-content

As you address these items, please take this last chance to review your reference list to ensure that it is complete and correct. If you have cited papers that have been retracted, please include the rationale for doing so in the manuscript text, or remove these references and replace them with relevant current references. Any changes to the reference list should be mentioned in the cover letter that accompanies your revised manuscript.

In addition to these revisions, you may need to complete some formatting changes, which you will receive in a follow up email. A member of our team will be in touch with a set of requests shortly. If you do not receive a separate email within a few days, please assume that checks have been completed, and no additional changes are required.

We expect to receive your revised manuscript within two weeks.

To submit your revision, please go to https://www.editorialmanager.com/pbiology/ and log in as an Author. Click the link labelled 'Submissions Needing Revision' to find your submission record. Your revised submission must include the following:

- a cover letter that should detail your responses to any editorial requests, if applicable, and whether changes have been made to the reference list

- a Response to Reviewers file that provides a detailed response to the reviewers' comments (if applicable, if not applicable please do not delete your existing 'Response to Reviewers' file.)

- a track-changes file indicating any changes that you have made to the manuscript.

NOTE: If Supporting Information files are included with your article, note that these are not copyedited and will be published as they are submitted. Please ensure that these files are legible and of high quality (at least 300 dpi) in an easily accessible file format. For this reason, please be aware that any references listed in an SI file will not be indexed. For more information, see our Supporting Information guidelines:

https://journals.plos.org/plosbiology/s/supporting-information

*Published Peer Review History*

Please note that you may have the opportunity to make the peer review history publicly available. The record will include editor decision letters (with reviews) and your responses to reviewer comments. If eligible, we will contact you to opt in or out. Please see here for more details:

https://plos.org/published-peer-review-history/

*Press*

Should you, your institution's press office or the journal office choose to press release your paper, please ensure you have opted out of Early Article Posting on the submission form. We ask that you notify us as soon as possible if you or your institution is planning to press release the article.

*Protocols deposition*

To enhance the reproducibility of your results, we recommend that if applicable you deposit your laboratory protocols in protocols.io, where a protocol can be assigned its own identifier (DOI) such that it can be cited independently in the future. Additionally, PLOS ONE offers an option for publishing peer-reviewed Lab Protocol articles, which describe protocols hosted on protocols.io. Read more information on sharing protocols at https://plos.org/protocols?utm_medium=editorial-email&utm_source=authorletters&utm_campaign=protocols

Please do not hesitate to contact me should you have any questions.

Sincerely,

Melissa

Melissa Vazquez Hernandez, Ph.D.

Senior Editor

mvazquezhernandez@plos.org

PLOS Biology

# Author comment: Author response to Decision Letter 3

**DOI:** 10.1371/journal.pbio.3003977.r007

11 Aug 2026

Attachment Submitted filename: Response_to_Reviewers_auresp_3.docx

# Editor report: Decision Letter 3

**DOI:** 10.1371/journal.pbio.3003977.r008

**Authors:** Melissa Vazquez Hernandez

12 Aug 2026

Dear Nishad,

Thank you for the submission of your revised Research Article "Polymorphisms in a bacterial signaling pathway alter evolutionary routes of antibiotic resistance in Escherichia coli" for publication in PLOS Biology. On behalf of my colleagues and the Academic Editor, Arjan de Visser, I am pleased to say that we can in principle accept your manuscript for publication, provided you address any remaining formatting and reporting issues. These will be detailed in an email you should receive within 2-3 business days from our colleagues in the journal operations team; no action is required from you until then. Please note that we will not be able to formally accept your manuscript and schedule it for publication until you have completed any requested changes.

Please take a minute to log into Editorial Manager at http://www.editorialmanager.com/pbiology/, click the "Update My Information" link at the top of the page, and update your user information to ensure an efficient production process.

PRESS

We frequently collaborate with press offices. If your institution or institutions have a press office, please notify them about your upcoming paper at this point, to enable them to help maximise its impact. If the press office is planning to promote your findings, we would be grateful if they could coordinate with biologypress@plos.org. If you have previously opted in to the early version process, we ask that you notify us immediately of any press plans so that we may opt out on your behalf.

We also ask that you take this opportunity to read our Embargo Policy regarding the discussion, promotion and media coverage of work that is yet to be published by PLOS. As your manuscript is not yet published, it is bound by the conditions of our Embargo Policy. Please be aware that this policy is in place both to ensure that any press coverage of your article is fully substantiated and to provide a direct link between such coverage and the published work. For full details of our Embargo Policy, please visit http://www.plos.org/about/media-inquiries/embargo-policy/.

Thank you again for choosing PLOS Biology for publication and supporting Open Access publishing. We look forward to publishing your study.

Sincerely,

Melissa

Melissa Vazquez Hernandez, Ph.D., Ph.D.

Senior Editor

PLOS Biology

mvazquezhernandez@plos.org
